# Long-range-interacting topological photonic lattices breaking channel-bandwidth limit

**DOI:** 10.1038/s41377-024-01557-4

**Published:** 2024-09-02

**Authors:** Gyunghun Kim, Joseph Suh, Dayeong Lee, Namkyoo Park, Sunkyu Yu

**Affiliations:** 1 Department of Electrical and Computer Engineering, Intelligent Wave Systems Laboratory, Seoul National University, Seoul, 08826 Korea; 2 Department of Electrical and Computer Engineering, Photonic Systems Laboratory, Seoul National University, Seoul, 08826 Korea

**Keywords:** Optical physics, Photonic crystals

## Abstract

The presence of long-range interactions is crucial in distinguishing between abstract complex networks and wave systems. In photonics, because electromagnetic interactions between optical elements generally decay rapidly with spatial distance, most wave phenomena are modeled with neighboring interactions, which account for only a small part of conceptually possible networks. Here, we explore the impact of substantial long-range interactions in topological photonics. We demonstrate that a crystalline structure, characterized by long-range interactions in the absence of neighboring ones, can be interpreted as an overlapped lattice. This overlap model facilitates the realization of higher values of topological invariants while maintaining bandgap width in photonic topological insulators. This breaking of topology-bandgap tradeoff enables topologically protected multichannel signal processing with broad bandwidths. Under practically accessible system parameters, the result paves the way to the extension of topological physics to network science.

## Abstract (web summary)

Topological lattices with long-range interactions enable increased Chern numbers and wide bandgaps, achieving defect-robust multichannel processing.

## Graphical abstract

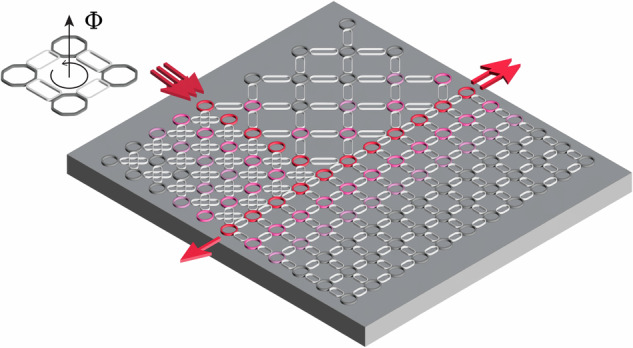

## Introduction

Nontrivial topological states of electrons^1–3^ have inspired their analogy in photonics^4^, by employing nonreciprocal materials^5,6^, lattice engineering^7,8^, pseudomagnetic fields^9–11^ and synthetic-dimensional systems^12^. The major focus of these studies is to achieve optical functionalities immune to defects, such as guiding^13^, multiplexing^14^, lasing^15^, frequency conversion^16^ and memory^17^. In these applications, the information capacity of noise-immune signal processing is proportional to the number of topological channels^13,18^, which is determined by topological charges of subsystems. Because larger topological charges require more singularities—Dirac or quadratic points—in topological gap opening, a larger number of topological channels are usually accessible at higher frequency bands having rapid phase evolutions. Therefore, relatively narrow higher-order bandgaps^19^ lead to the trade-off between the number of topological channels and channel bandwidths. Overcoming this trade-off to achieve multi-channel topological circuits is still a challenging issue.

A breakthrough could be found in a previously overlooked degree of freedom in topological systems—long-range connectivity, especially, the systems possessing stronger long-range interactions than short-range ones. In network science, it was recently proved that a Laplacian corresponding to an arbitrary fully-connected network can provide a free-form level statistics, demonstrating the inverse design of bandgaps with designed gap widths^20^. Although such a network-based approach did not handle topological phenomena, the design freedom obtained from substantial long-range connectivity implies the potential for topological photonic systems, where the tradeoff between topological channels and channel bandwidths is alleviated. Although substantial efforts have been applied to exploit long-range interactions for topological phenomena, such as Kagome lattices^21^, quasicrystals^22,23^ and synthetic dimensional systems^24^, most of the studies assume smaller long-range interactions than short-range ones, leaving behind unexplored design freedom with stronger long-range connectivity.

Here, we exploit substantial long-range interactions to break channel-bandwidth limit in topological photonics. As an extreme scenario, we focus on long-range connectivity in the absence of nearest-neighbor interactions. To analyze such long-range connectivity, we propose the lattice overlap model: the overlap of unit lattices having nearest-neighbor interactions. This lattice overlap strategy provides the design freedom for achieving arbitrary Chern number while preserving the bandgap width, as demonstrated in the Hofstadter model example. Using the system parameters available in conventional silicon photonics, we demonstrate the incoherent optical functionality—designed manipulation of multichannel light with random phases and amplitudes—which is robust to various types of disorder originating from manufacturing defects. Achieving noise-immune signal processing with enhanced information capacity, our design principle paves the way to implementing topological phenomena in complex networks.

## Results

### Long-range connectivity

**Fig. 1:**
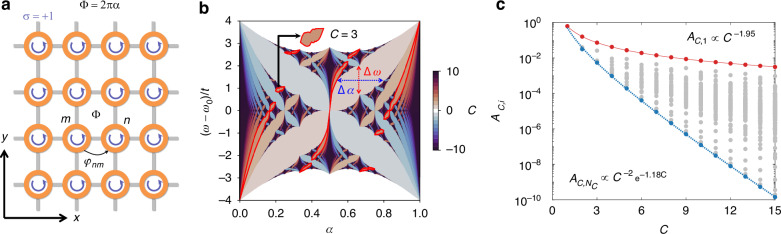
Multichannel signal transport performance in the Hofstadter model. **a** A schematic of a coupled-resonator square lattice with nearest-neighbor interactions between pseudospin modes (*σ* = +1). **b** The Hofstadter butterfly, representing the gap Chern numbers *C* with different colors. An example of the wing segment for *C* = 3 is illustrated with the red outlines. The color bar represents *C*. **c** The tradeoff between *C* and *A*_*C*,*i*_. The red and blue lines denote the *C*-*A*_*C*,*i*_ relationships for the largest and smallest wings, respectively. The gray dots depict the areas of intermediate-sized wings

Before examining long-range connectivity, we revisit the Hofstadter model^25,26^, which is a building block of our system. In integrated photonics, the model can be realized with the coupled-resonator lattice of which the unit resonator supports pseudospin modes^10,12^ (Fig. 1a). When the opposite pseudospins are decoupled, the effective tight-binding Hamiltonian of the lattice for each pseudospin is

1 $$H=\omega_{0}\sum\limits_{n,\sigma}a_{n,\sigma}^{\dagger}a_{n,\sigma} - t \sum\limits_{\langle m,n\rangle,\sigma}e^{-i\sigma\varphi_{mn}}a_{m,\sigma}^{\dagger}a_{n,\sigma}$$

where *ω*_0_ is the resonance frequency of each resonator, ${a}_{n,\sigma }^{\dagger }$ and *a*_*n*,*σ*_ are the creation and annihilation operators of the pseudospin mode *σ* = ±1 at the *n*th site, respectively, *t* and *φ*_*mn*_ are the amplitude and phase of the interaction between the *m*th and *n*th sites, respectively, and 〈*m*, *n*〉 denotes the summation indices over all the nearest-neighbor resonator pairs. To realize broken time-reversal symmetry for a pseudospin mode *σ* = +1, nearest-neighbor interactions are designed to impose the synthetic flux Φ = +2π*α* on the photons during a counterclockwise rotation around each unit cell. The flux for the square lattice (Fig. 1a) is achieved with the following hopping phases using the Peierls substitution^27^:

2 $$\varphi_{mn}=\pm 2\pi\alpha m_{1}\delta_{m_{1},n_{1}}\delta_{m_{2},n_{2} \pm 1}$$

where (*m*_1_, *m*_2_) and (*n*_1_, *n*_2_) are the integer indices denoting the positions of the *m*th and *n*th sites, respectively, and *δ*_*a*,*b*_ is the Kronecker delta function. The designed hopping phase can be implemented with non-resonant waveguide loops^10^, phase delays inside strongly coupled resonators^28^, or spatiotemporal modulations for synthetic frequency dimensions^29^. We analyze the non-resonant waveguide loops in Materials and methods and Supplementary Note S1, which allow arbitrary hopping strengths and phases independent of the spatial distance between the resonator sites.

The topological invariant determining the number of topologically protected edge modes^30^ in the Hofstadter model is the gap Chern number—the sum of the Chern numbers of the bands below a specific gap^4,31^. For the flux with the rational number *α* = *p*/*q* for the coprime positive integers *p* and *q*, the gap Chern number *C*_*l*_ of the *l*th gap is obtained with the Thouless-Kohmoto-Nightingale-den Nijs (TKNN) formula^2^, as follows:

3 $$C_{l}(p,q)\equiv lp^{-1} ({\bmod}\, q)\,\,{\rm{and}}\,\,-q/2\leq C_{l}(p,q)\leq q/2$$

where *C*_*l*_(*p*,*q*) is well-defined except for gap closing with even *q* and *l* = *q*/2. The colored Hofstadter butterfly^32^ shown in Fig. 1b illustrates the gap Chern numbers for the rational *α* and frequency *ω* (See Materials and methods and Supplementary Note S2).

According to Eq. (3), *C*_*l*_ can have an arbitrary integer by controlling the flux parameters *p* and *q*, and the bandgap number *l*, allowing any numbers of topologically protected edge modes within the frequency range of *ω*_0_ – 4*t* < *ω* < *ω*_0_ + 4*t*. However, increasing the number of edge modes substantially decreases the bandgap width (Δ*ω* in Fig. 1b) and the allowed range of synthetic fluxes (Δ*α* in Fig. 1b). This tradeoff between *C*_*l*_ and the parameter ranges Δ*ω* and Δ*α* limits the signal transport performance in multichannel applications. For example, the operation bandwidth of the edge modes is restricted by Δ*ω*, and the allowed range of Δ*α* determines the robustness of the flux, which is very sensitive to optical phases.

To quantify the tradeoff relation, first we investigate the number of butterfly wings—or topological bandgaps—*N*_*C*_ for a specific gap Chern number *C* ≡ *C*_*l*_: *N*_*C*_ = *φ*(1) + ··· + *φ*(2 | *C* | ) for the Euler’s phi function *φ*(*n*) (Supplementary Note S3). The performance of *C*-channel signal transport using topologically protected edge modes can then be characterized by the area *A*_*C*,*i*_ of the *i*th wing (*i* = 1, 2, …, *N*_*C*_) for each *C*, where

4 $$A_{C,i}=\int_{{\alpha_{C,i}}^{\rm{L}}}^{{\alpha_{C,i}}^{\rm{U}}}\Delta \omega(\alpha)d\alpha$$

Δ*ω*(*α*) is the bandgap width at *α*, and *α*_*C*,*i*_^U^ and *α*_*C*,*i*_^L^ are the upper and lower bounds of the wing, respectively, satisfying the gap closing at Δ*ω*(*α*_*C*,*i*_^U^) = Δ*ω*(*α*_*C*,*i*_^L^) = 0. We numerically calculate *A*_*C*,*i*_ for *C* ≤ 15 by approximating each wing as a 60-gon (Supplementary Note S3).

Figure 1c shows the tradeoff relation between the channel number *C* and the signal transport performance *A*_*C*,*i*_. Notably, we reveal the *i*-dependent transition between the power law and exponential distributions between *C* and *A*_*C*,*i*_. First, the largest wings (*i* = 1) satisfy the power law *A*_*C*,1_ ∝ *C*^–1.95^ (red solid line in Fig. 1c). This quadratic scaling originates from *C*-dependencies of Δ*α* = *α*_*C*,*i*_^U^ – *α*_*C*,*i*_^L^ and Δ*ω*: Δ*α* is equal to *C*^–1^/2, and Δ*ω* is approximately proportional to *C*^–1^ due to the convergence to the Landau levels^33^ as *α* goes to 0 or 1. In contrast, the smallest wings (*i* = *N*_*C*_) show a distinct scaling property—a power law with an exponential cutoff (${A}_{C,{N}_{C}}$ ∝ *C*^−2^*e*^–1.18*C*^, blue dotted line in Fig. 1c). While the power-law component in ${A}_{C,{N}_{C}}$ is attributed to the scaling Δ*α* ~ *C*^–2^ for the smallest wings, the exponential cutoff originates from the fractal nature of the Hofstadter butterfly^25^, because the smallest wings correspond to higher-order recursive steps of the butterfly fractal. Therefore, the recursive change of Δ*ω* through the fractal evolution leads to the exponential distribution. We note that regardless of the bandgap index *i*, the signal transport performance *A*_*C*,*i*_ is severely restricted by *C*.

To overcome the limit of the signal transport performance *A*_*C*,*i*_, we exploit long-range connectivity preserving discrete translational symmetry. We focus on a class of the long-range connectivity that can be decomposed into the decoupled overlap of the translated lattices having only nearest-neighbor interactions. For example, overlapping the identical lattices with in-plane translation allows for the coexistence of their eigenstates. Because Δ*ω* and Δ*α* maintain their values of an individual lattice due to decoupling, the increase of the gap Chern number *C* is expected while preserving the performance figure *A*_*C*,*i*_. Importantly, the vertical stacking of identical two-dimensional (2D) systems, which has recently become a substantial idea for achieving high Chern numbers in magnetic topological insulator films^34–37^, also belongs to a class of our long-range connectivity under the presence of the inter-layer interactions between the composing lattices. However, these previous studies on vertical stacking^34–37^ also assume smaller long-range (or inter-layer) interactions than short-range (or intra-layer) ones. Furthermore, realizing stacking structures within photonic circuit geometry poses substantial challenges, for example, in achieving the gauge field in inter-layer interactions.

**Fig. 2:**
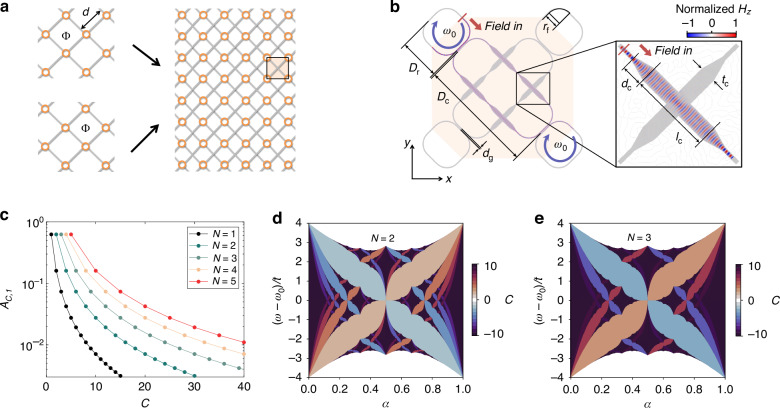
Overlapped Hofstadter lattices for long-range connectivity. **a** Schematics of the two decoupled square lattices having nearest-neighbor interactions (left) and the overlapped lattice having only next-nearest-neighbor interactions (right). Orange circles and gray lines denote the site resonators and non-resonant waveguide couplers. The lattice constant of each composing lattice is *d*. The black box on the overlapped lattice indicates the crossing between long-range interactions. **b** The silicon waveguide implementation^38^ of the black box region in (**a**). The inset shows the FDTD simulation result for the waveguide crossing region. *D*_r_, *D*_c_, *d*_g_, *d*_c_*, r*_f_, *l*_c_ and *t*_c_ represent geometric parameters (Materials and methods for their values). **c** The maximum wing area *A*_*C*,1_ with respect to the gap Chern number *C* for different numbers of the overlaps *N*. The Hofstadter butterflies for *N* = 2 (**d**) and *N* = 3 (**e**). Color bars represent *C*

To employ the overlap of lattices, we apply the following two criteria for real implementation. First, the sites and connections of the lattices should not be overlapped to maintain the band properties of the original lattices. Second, the pointwise crossings between the connections are allowed by employing the waveguide crossing structures with negligible crosstalks and losses. We demonstrate our proposal by overlapping two lattices as an example. Figure 2a shows two decoupled square lattices possessing nearest-neighbor interactions with the same lattice constant *d*. In each lattice, we impose the hopping phase of the Hofstadter model with the same *α*. The lattices are 45° rotated from the original Hofstadter lattice (Fig. 1a) while maintaining connectivity. When the lattices are overlapped in the 2D plane by translating a lattice *d*/2^1/2^ along the *x*-axis, the obtained overlapped square lattice has next-nearest-neighbor interactions with nearest-neighbor decoupling, realizing long-range connectivity.

The Hamiltonian of the overlapped lattice is given by the direct sum as:

5 $$H=H_{1}\oplus H_{2}$$

where *H*_1_ and *H*_2_ are the Hamiltonians of the Hofstadter model represented by Eq. (1), corresponding to the top and bottom square lattices in Fig. 2a, respectively. Because *H*_1_ and *H*_2_ possess identical band structures, the Hamiltonian *H* leads to the degeneracy of the original bands. The generalization of the two-lattice overlap toward the overlap of an arbitrary number of lattices can also be described with the direct sum: *H* = ⨁_*n*_*H*_*n*_.

The hurdle in realizing the overlapped lattice is the crossings between long-range interactions (black box in Fig. 2a). To resolve this issue, we employ a waveguide crossing design to a non-resonant waveguide loop (Fig. 2b). By applying conventional system parameters in silicon photonics^38^, we validate the suppression of unwanted crosstalk with the finite-difference time-domain (FDTD) full-wave analysis (See Materials and methods and Supplementary Note S4). The simulation result shows that the device provides the long-range interaction strength an order of magnitude larger than radiation loss (−21 dB) and nearest-neighbor interactions (−28 dB).

To demonstrate our proposal, we calculate the maximum wing area *A*_*C*,1_ for different numbers of the overlapped lattices (Fig. 2c). The increase of *A*_*C*,1_ is directly proportional to the number of overlaps *N*, while preserving the power law behavior with the same exponent. Figure 2d, e illustrate the Hofstadter butterflies of two- and three-folded lattices, respectively, proving that the gap Chern numbers of an *N*-folded lattice domain are *N* times the original gap Chern number due to the degeneracy of the decoupled lattice overlap. We also note that the overlap of disparate lattices allows for any gap Chern number other than the multiples of *N* (Supplementary Note S5).

### Topologically nontrivial edge modes

**Fig. 3:**
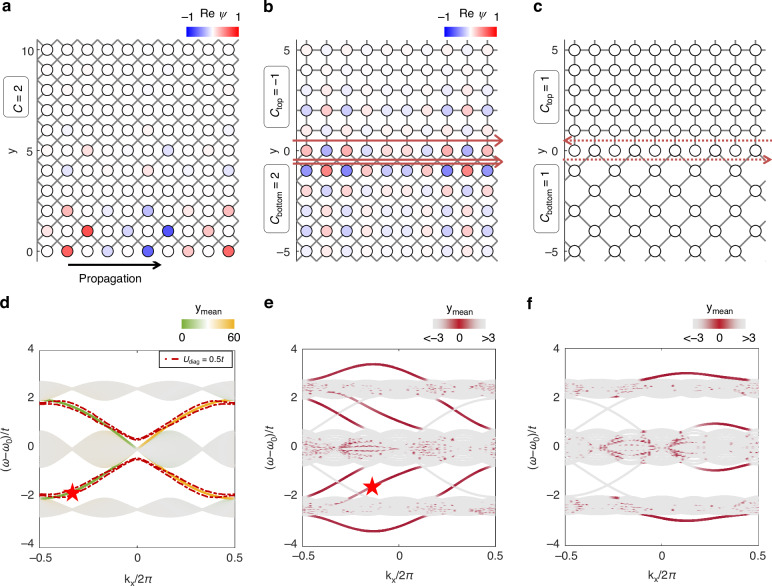
Edge mode analysis. **a** Field profile of the topologically nontrivial edge mode in the overlapped lattice. The domain has *α* = 1/3 for each sublattice, leading to *C* = 2 in the lower gap. The black arrow indicates the propagating direction of the mode. **b** Field profile of the topologically nontrivial edge mode at the interface between two overlapped lattices. The adjacent domains have inhomogeneous Chern numbers (*C*_bottom_ = 2 and *C*_top_ = −1). **a**, **b** denote the star markers in (**d**, **e**), respectively. **c** The absence of the topologically nontrivial edge mode at the interface between two overlapped lattices with homogeneous Chern numbers (*C*_bottom_ = *C*_top_ = 1). **d**−**f** The band structures of the systems in (**a**−**c**), respectively. *k*_*x*_ denotes the *x*-axis component of the reciprocal vector in the magnetic Brillouin zone. *y*_mean_ denotes the *y*-axis component of the center-of-mass of the corresponding modes. The dashed lines in (**d**) represent the ensemble averaged dispersions under 500 realizations of uniformly random diagonal disorder [–*U*_diag_,+*U*_diag_] on the resonance frequency, where *U*_diag_ = 0.5*t*. All the results are obtained with the tight-binding equation with the supercell technique (Supplementary Note S6)

To explore nontrivial topological natures of overlapped lattices, we investigate the edge modes at lattice boundaries. We compare three different configurations: the surface of a bulk (Fig. 3a) and the interfaces between the bulks having different (Fig. 3b) and same *C*’s (Fig. 3c). To obtain dispersion relations, we employ the one-dimensional (1D) ribbon geometry, which is infinite along the *x*-axis with the finite *y*-axis thickness.

Figure 3a shows a finite bulk domain of a doubly overlapped lattice. A sublattice composing the domain has the synthetic flux of *α* = 1/3, leading to two bandgap openings with *C* = ± 1. The corresponding band structure is shown in Fig. 3d (Supplementary Note S6 for its calculation). The overlapped lattice composed of two identical sublattices provides doubly degenerate edge modes, which are apparent when diagonal disorder is applied (red dashed lines in Fig. 3d).

Figure 3b, e, c, f show the *C*-dependency of edge modes by examining the overlapped lattices having inhomogeneous and homogeneous *C* profiles, respectively. In Fig. 3b, we impose different synthetic fluxes on the bottom (*α* = 1/3) and top (*α* = −1/3) lattices. Due to the symmetric profiles of the Hofstadter butterflies for the overlapped lattices (Fig. 2d, e), the bandgaps of the bottom and top lattices fall within the same frequency range for the same |*α*|. Therefore, the given configuration in Fig. 3b facilitates the full use of the bandgap for signal bandwidths. Meanwhile, the gap Chern numbers of the bottom and top lattices at the first bandgap are disparate: *C*_bottom_ = 2 and *C*_top_ = −1, respectively. Therefore, three topologically protected edge modes are obtained from |*C*_bottom_ – *C*_top_ | = 3 (Fig. 3e). In contrast, *C*-homogeneous bulks in Fig. 3c, which are achieved with *α* = 1/3 at both bottom and top overlapped lattices, lead to the absence of topologically nontrivial edge modes (Fig. 3f).

Edge mode dispersions in Fig. 3e, f can be illustrated through the co- and counter-directional coupling between the edge modes in the isolated bulks, where the number of the modes is directly determined by *C*. For example, the interface in Fig. 3b can be understood as the junction of three planes: a plane with *C* = –1 and two planes with *C* = +1, resulting in the coupling of three co-propagating edge modes (solid arrows in Fig. 3b). On the contrary, the structure with homogeneous *C* in Fig. 3c results in the counter-propagating edge modes (dashed arrows in Fig. 3c), leading to the stark anti-crossing and the following transition from topologically nontrivial to trivial interface states (Fig. 3f).

### Scattering analysis

**Fig. 4:**
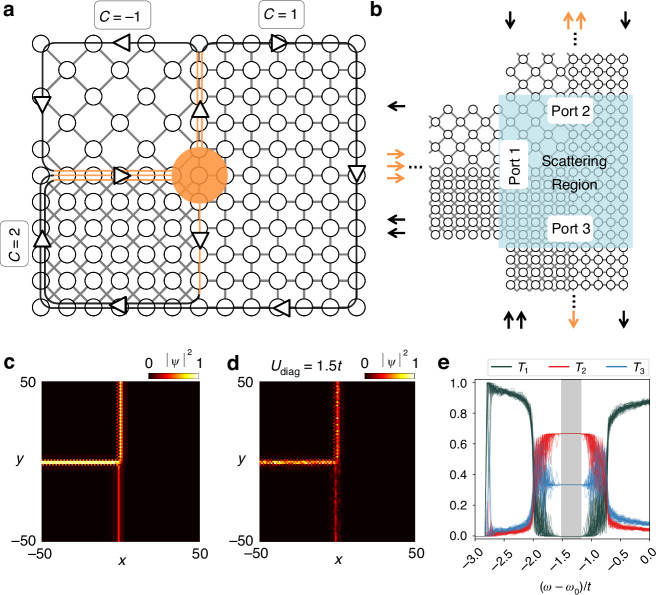
Incoherent multichannel beam splitting. **a** A schematic of the scattering region. Three domains possess *α* = ±1/3 to support the gap Chern number ±1 at the shared bandgap range. A 101 × 101 scattering region is assumed for the analysis in (**c**−**e**). **b** The scattering region with semi-infinite ports for applying the scattering matrix method. The orange arrows represent the centered edge modes of each port. The black arrows denote the outer edge modes resulting from finite widths of the ports. The incoherent transmittance is proportional to the number of centered edge modes. **c** Intensity profile for an incoherent light at the frequency *ω* = *ω*_0_ − 1.5*t*. The input is obtained with 100 random linear combinations of the input edge modes. **d** Intensity profile against diagonal disorder. The resonance frequency of each site in the scattering region is perturbed uniformly between [−*U*_diag_,+*U*_diag_]. **e** The transmittances for the incoherent incidence against diagonal disorder. The transmittance curves for 50 realizations of diagonal disorder are overlaid. *U*_diag_ = 1.5*t* in (**d**, **e**). The shaded region in (**e**) denotes the frequency range of perfect beam splitting operation against disorder

To confirm the enhanced information capacity in signal transport through overlapped topological lattices, we analyze the scattering of edge modes. We examine the system that includes three interfaces between the overlapped lattices, thereby composing an edge-mode beam splitter (Fig. 4a, b). All the sublattices for the lattice overlap are designed with *α* = ±1/3, where the overlap derives different numbers and chirality of edge modes. To examine the scattering at the junction of the topological beam splitter, we calculate the frequency-dependent scattering matrix *S*_*ij*_(*ω*) between the *i*th and *j*th edge modes within the bandgap, using the open-source Python package Kwant^39^ (See Materials and methods and Supplementary Note S7). To examine the scattering at the junction of the topological beam splitter, we calculate the frequency-dependent scattering matrix between the edge modes within the bandgap, using the open-source Python package Kwant^39^ (See Materials and methods and Supplementary Note S7).

To fully exploit the superior performance of the overlapped lattices—large *A*_*C*,*i*_ with broader bandgaps and multiple edge modes—we focus on topologically protected wave functionalities applied to spatio-temporally incoherent light sources. The light source is defined by the superposition of the edge modes having uniformly random amplitudes and phases. To quantify the energy flow throughout the *k*th port (*k* = 1, 2, 3), we define incoherent transmittance *T*_*k*_ as the average of the squares of the scattering coefficients between port 1 and port *k*:

6 $$T_{k}(\omega)=\frac{1}{N_{{\rm{in}},1}}\sum\limits_{i=1}^{N_{{\rm{out}},k}}\sum\limits_{j=1}^{N_{{\rm{in}},1}}|S_{ij}(\omega)|^{2}$$

where *N*_in,1_ is the number of the input modes at port 1, *N*_out,*k*_ is the number of the output modes throughout port *k*. Because the centered edge modes (orange arrows in Fig. 4b) barely overlap with the outer modes (black arrows in Fig. 4b), the sub-scattering matrix consisting of the centered edge modes is almost unitary.

From the unitary sub-scattering matrix, *T*_*k*_ in the frequency region supporting the centered edge modes is solely determined by the number of centered edge modes under the incoherent incidences (Fig. 4c), regardless of the phase matching condition at the splitter junction. Due to topologically nontrivial natures, the beam splitting is protected under different forms of disorder: diagonal (Fig. 4d) and hopping phase disorders (Supplementary Note S8). As shown in Fig. 4e, the beam splitting is maintained over the bandgap range with backscattering suppression, demonstrating incoherent functionality for multichannel integrated photonics for the first time.

## Discussion

We have exploited the long-range connectivity to break channel-bandwidth limit in topological photonics. By quantitatively analyzing the Hofstadter butterfly, we have revealed the power laws between the gap Chern numbers and the area of the wings. The observed quadratic scaling underlies the necessity of breaking the tradeoff between the bandgap and Chern number. We have modeled the long-range interactions as the overlap of the lattices having nearest-neighbor interactions, which enables the breaking of the tradeoff as demonstrated in the band analysis. Employing this feature, we have demonstrated the defect-robust beam splitting functionality applicable to incoherent light. All the system parameters are devised within the regime of conventional silicon photonics technology.

In evaluating the performance of our overlapped lattice model, it is necessary to examine the relationship among device bandwidth and lattice sizes (Supplementary Note S9). Notably, the spectral bandwidth of the Hofstadter model is primarily governed by the hopping constant *t*, which determines the bandgap width (Supplementary Fig. S14). However, the variation of lattice sizes due to the lattice overlap eventually affects the device bandwidth. As illustrated in Supplementary Fig. S15a, our overlap model increases the distance between the connected resonators even under an ideal condition—namely, neglecting the spatial footprint of waveguide crossing structures. Furthermore, to guarantee sufficiently large spectral bandwidths during the hopping process, we employ an adiabatic design for waveguide crossing structures with slowly-varying shapes (Fig. 2b), which leads to more degraded integration level of the lattice (Supplementary Fig. S15c). Consequently, to maintain the original size of the Hofstadter lattice during the practical overlap process, we need to employ a more compact waveguide crossing design, for example, using impedance matching techniques, which is typically suitable for narrowband operations. In this context, designing a waveguide crossing structure that is compact yet offers a wide bandwidth will be a significant challenge in fully leveraging the benefits of our lattice overlap model.

Besides the proposed silicon-waveguide implementation, alternative platforms are available for realizing the overlapped lattices. First, optical resonators under spatio-temporal modulations can be applied to construct the overlapped lattices across both spatial and synthetic dimensions^40^, which enables multimodal frequency conversion. Notably, the interaction along the synthetic dimension allows for implementing long-range interactions without the crossing structure in the real space. Second, electronic circuits^41^ provide more practical frameworks to the proof-of-concept experiments for topological overlapped lattices, as demonstrated in the realization of long-range interactions in circuitry^42,43^. The increased design freedom from three-dimensional wiring allows for implementing any graph networks representing linear Hamiltonians, including the overlapped lattice model examined in this work. Furthermore, coupled non-Hermitian resonators^44^ and their coupling with nonsymmetric internal structures^45,46^ are promising platforms to extend our overlapped lattice model into non-Hermitian topological photonics. The designed access to both spin modes using the internal structure^45–47^ is also an essential building block when time-reversal symmetry breaking is applied to resolve unwanted backscattering of the opposite spin^10,48^ with time-varying^9^ or magneto-optical media^6^.

The generality of lattice overlap modeling also inspires further studies. First, the overlap is applicable to other topological systems with different symmetries such as the Haldane^46^, higher-order^49^, or hyperbolic models^50^. In Supplementary Note S10, we show an example of the overlap strategy for the Haldane lattices, which can be implemented with our waveguide crossing design that supports negligible crosstalks and losses. Second, there exists an unexplored intermediate regime between our long-range-dominant overlapped lattices and the majority of other long-range topological models^51–53^, where the nearest-neighbor interactions are most significant. In Supplementary Note S11, we examine the competition between the nearest-neighbor and next-nearest-neighbor interactions on the Hofstadter overlapped lattices, which was studied in the nearest-neighbor-dominant regime for the Haldane model^46^. The result in Supplementary Note S11 shows the phase transition of band structures due to the mixing of the degenerate bands. Further investigation on the topological phases under the coexistence of even higher long-range interactions paves the way toward the realization of complex networks in the realm of topological physics.

## Materials and methods

### Tight-binding description of waveguide-loop coupling

We implement the overlapped lattice composed of ring resonators, which are pair-wisely coupled via off-resonant waveguide loops. A pair of coupled ring resonators is described by the following tight-binding Hamiltonian^10,50^:

7 $$H=\omega_{0}(a^{\dagger}a+b^{\dagger}b)-\frac{1}{\tau}(e^{-i\varphi}a^{\dagger}b+e^{i\varphi}b^{\dagger}a)$$

where *a*^†^, *a*, *b*^†^, and *b* are the creation and annihilation operators of the resonator ‘a’ and ‘b’, *ω*_0_ is the resonance frequency, *τ* is the energy leakage rate from each resonator to the evanescently coupled waveguide, and *φ* is the tunable phase shift obtained through the waveguide loop (Supplementary Note S1 for derivation from the temporal coupled mode theory).

### Chern number illustration in the Hofstadter Butterfly

We color the Hofstadter butterflies in Figs. 1c, 2d, e on a pixel-by-pixel basis with a 1440 × 1920 resolution. For each *α* = *i*/1920 (0 ≤ *i* < 1920), we numerically calculate the band structure of the Hofstadter Hamiltonian of Eqs. (1) and (2), discretizing the repeated part of the magnetic Brillouin zone as 20 × 20 points (Supplementary Note S2 for details). We determine the Chern number using the TKNN formula, Eq. (3), for each of the 1440 frequencies within the range (*ω*_0_ − 4*t*, *ω*_0_ + 4*t*).

### Silicon photonics design of the overlapped lattice

We perform the FDTD full-wave analysis using Tidy3D^54^ on the cross-waveguide structure (Fig. 2b), which is the building block of the long-range interacting overlapped lattice. Assuming silicon slab waveguides (*ε*_*r*,Si_ = 12.11) on a silicon dioxide substrate (*ε*_*r*,SiO2_ = 2.10), we employ the geometrical parameters: *D*_r_ = 16 μm, *D*_c_ = 44 μm, *d*_g_ = 100 nm, *d*_c_ = 2.5 μm, *r*_f_ = 5.5 μm, *l*_c_ = 9.3 μm, *t*_c_ = 1.9 μm, the waveguide width *w*_wg_ = 0.5 μm, and height *h*_wg_ = 0.22 μm (Fig. 2b). Through the transfer matrix analysis, we obtain the long-range coupling coefficient *t* = 40 GHz with enough suppression of the crosstalk (*κ*′ = 1.6 GHz) and system loss (*κ*_ext_ = 3.5 GHz) at the operating wavelength *λ*_0_ = 1551.5 nm (Supplementary Note S4 for details).

### Scattering matrix analysis

To extract the scattering matrix between the edge modes at the center junction in Fig. 4a, we extend the ports of the system to support the edge modes propagating from and to infinitely away while satisfying impedance matching. Three semi-infinite ports (Fig. 4b) are constructed by repeating the supercells of the overlapped lattices (Supplementary Note S6). We utilize the open-source python package Kwant^39^ to solve the propagating edge modes and scattering matrices (Supplementary Note S7 for details).

## Supplementary information

Supplementary Information for “Long-range-interacting topological photonic lattices breaking channel-bandwidth limit”

## Data Availability

The data that support the plots and other findings of this study are available from the corresponding author upon request.

## Code availability

All code developed in this work will be made available from the corresponding author upon request.
