# Author Correction: Long-range-interacting topological photonic lattices breaking channel-bandwidth limit

**DOI:** 10.1038/s41377-024-01715-8

**Published:** 2025-01-04

**Authors:** Gyunghun Kim, Joseph Suh, Dayeong Lee, Namkyoo Park, Sunkyu Yu

**Affiliations:** 1 Department of Electrical and Computer Engineering, Intelligent Wave Systems Laboratory, Seoul National University, Seoul, 08826 Korea; 2 Department of Electrical and Computer Engineering, Photonic Systems Laboratory, Seoul National University, Seoul, 08826 Korea

**Keywords:** Optical physics, Photonic devices

Correction to: *Light: Science & Applications* 10.1038/s41377-024-01557-4, published online 02 September 2024

After publication of this article^1^, we noticed that Fig. 2d and Fig. 2e were swapped in the process of generating editable figures by the authors after peer review. Because the Chern number increases with increasing *N*, the Hofstadter butterfly for *N* = 3 should be displayed with a darker color than that of *N* = 2 according to our colormap.

**Fig. 1:**
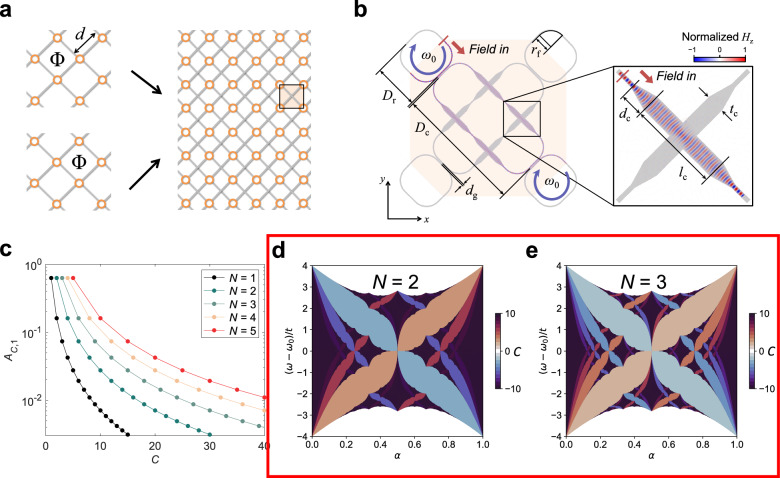
**Initially published version of Fig. 2**, where **d** and **e** are swapped.

**Fig. 2:**
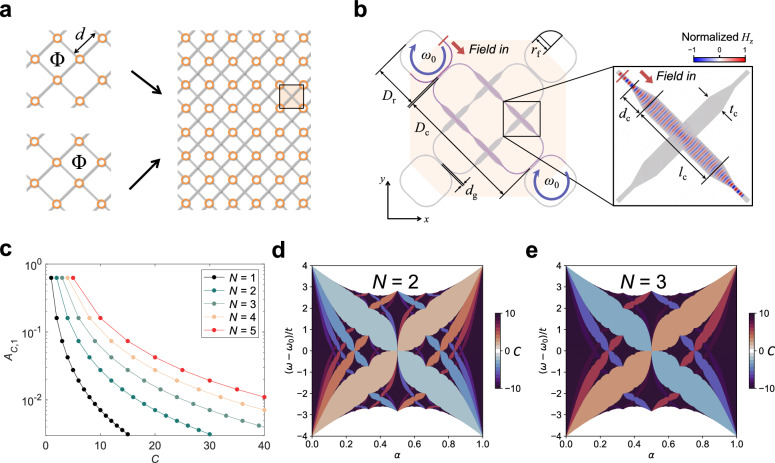
**Corrected version of Fig. 2**. With higher Chern numbers, the butterfly is darker in panel **e** (*N* = 3) than that in panel **d** (*N* = 2).

The original article has been corrected.
