# Supplementary material for: Long-range-interacting topological photonic lattices breaking channel-bandwidth limit
Source: Light Sci Appl. 2024 Sep 2;13:189. doi: 10.1038/s41377-024-01557-4 (PMC11366748; doi:10.1038/s41377-024-01557-4)
Supplement: Supplementary file 1 — Supplementary Information for “Long-range-interacting topological photonic lattices breaking channel-bandwidth limit” [file 41377_2024_1557_MOESM1_ESM.pdf]

Supplementary Information for "Long-range-interacting topological photonic lattices breaking channel-bandwidth limit"

Gyunghun Kim[1], Joseph Suh[1], Dayeong Lee[1], Namkyoo Park[2†], and Sunkyu Yu[1*]

[1]Intelligent Wave Systems Laboratory, Department of Electrical and Computer Engineering, Seoul National University, Seoul 08826, Korea

[2]Photonic Systems Laboratory, Department of Electrical and Computer Engineering, Seoul National University, Seoul 08826, Korea

E-mail address for correspondence: [†]nkpark@snu.ac.kr, [*]sunkyu.yu@snu.ac.kr

Note S1. Tight-binding description of waveguide-loop coupling

Note S2. Chern number illustration in the Hofstadter butterfly

Note S3. Butterfly wing area calculation

Note S4. System parameters of the overlapped lattice for silicon photonics

Note S5. Overlapping disparate lattices

Note S6. Calculating band structures using ribbon geometries

Note S7. Scattering matrix analysis

Note S8. Robustness against diagonal and hopping-phase disorders

Note S9. Performance limitations in lattice sizes and bandwidths

Note S10. Geometry of the overlapped Haldane lattice

Note S11. Competition between different orders of interactions in the overlapped lattice

Note S1. Tight-binding description of waveguide-loop coupling

We review the photonic tight-binding formulation of the ring resonators coupled via non-resonant waveguide loops (Fig. S1), which has been widely employed in topological photonics[1,2]. We consider only the pseudospin up (counterclockwise wave circulation) modes, neglecting the interactions between opposite pseudospins. The temporal coupled-mode theory (CMT) model[1,2] for Fig. S1 is:

$$\begin{cases} \dfrac{d}{dt}\begin{bmatrix}\psi_a\\ \psi_b\end{bmatrix} = \left(i\omega_0 - \dfrac{1}{\tau}\right)\begin{bmatrix}\psi_a\\ \psi_b\end{bmatrix} + \sqrt{\dfrac{2}{\tau}}\begin{bmatrix}s_2\\ s_3\end{bmatrix} \\ \begin{bmatrix}s_1\\ s_4\end{bmatrix} = \begin{bmatrix}s_2\\ s_3\end{bmatrix} - \sqrt{\dfrac{2}{\tau}}\begin{bmatrix}\psi_a\\ \psi_b\end{bmatrix} \\ \begin{bmatrix}s_1\\ s_4\end{bmatrix} = \begin{bmatrix}e^{-i\varphi_t}s_3\\ e^{-i\varphi_b}s_2\end{bmatrix} \end{cases}, \tag{S1}$$

where ψ_a and ψ_b represent the fields in each resonator, s_1, s_2, s_3, and s_4 denote the propagating fields at each position of the waveguide loop (Fig. S1), ω_0 is the resonant frequency of both resonators, τ is the lifetime of the resonance modes denoting the external energy leakage to the waveguide loop, and φ_t and φ_b are the phase changes of the light along the top and bottom parts of the waveguide loop, respectively. From Eq. (S1), we obtain

$$\frac{d}{dt}\begin{bmatrix}\psi_a\\ \psi_b\end{bmatrix} = i\omega_0\begin{bmatrix}\psi_a\\ \psi_b\end{bmatrix} + \frac{1}{\tau(1-e^{-i(\varphi_b+\varphi_t)})}\begin{bmatrix}1+e^{-i(\varphi_b+\varphi_t)} & 2e^{-i\varphi_b}\\ 2e^{-i\varphi_t} & 1+e^{-i(\varphi_b+\varphi_t)}\end{bmatrix}\begin{bmatrix}\psi_a\\ \psi_b\end{bmatrix}. \tag{S2}$$

The non-resonant condition of the waveguide loop is satisfied by assigning $\varphi_b + \varphi_t = (4m + 1)\pi$ for integer m. Under the additional assumption of $\varphi_b = \varphi + 2m\pi + \pi/2$ and $\varphi_t = -\varphi + 2m\pi + \pi/2$, we obtain

$$\frac{d}{dt}\begin{bmatrix}\psi_a\\ \psi_b\end{bmatrix} = i\begin{bmatrix}\omega_0 & -e^{-i\varphi}/\tau\\ -e^{i\varphi}/\tau & \omega_0\end{bmatrix}\begin{bmatrix}\psi_a\\ \psi_b\end{bmatrix}, \tag{S3}$$

which is expressed in the second quantization convention as[2]

$$H = \omega_0(a^\dagger a + b^\dagger b) - t(e^{-i\varphi} a^\dagger b + e^{i\varphi} b^\dagger a), \tag{S4}$$

where H is the Hamiltonian of the system, $a^\dagger$, $b^\dagger$, a, and b are the creation and annihilation operators of each resonator, respectively, and $t = 1/\tau$ denotes the coupling strength.

Extending the two-resonator system to a lattice shown in Fig. 1a in the main text is straightforward. A lattice of resonators is constructed by connecting the ring resonators via the non-resonant waveguide loops with the tailored offset δ (Fig. S1) to achieve the phase difference 2φ between the loop arms.

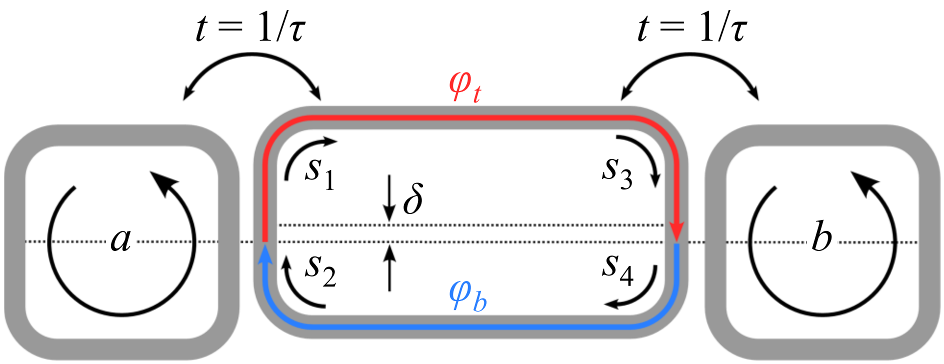

Fig. S1. Coupled mode theory for the tight-binding Hamiltonian. A schematic for the indirect coupling between two ring resonators with the tailored gauge field. δ denotes the displacement of the waveguide loop to induce the phase difference between the loop arms.

Note S2. Chern number illustration in the Hofstadter butterfly

The colored Hofstadter butterflies in Fig. 1c, 2d, and 2e of the main text illustrate the band structures and the gap Chern numbers of the Hamiltonian in Eq. (1) in the main text. We color the butterfly pixelwise: 1440 × 1920 pixels for the α- and ω-axes, respectively. Black and pastel-colored pixels correspond to the α-ω states in the bands and the gaps, respectively.

To determine the color of each point in the gaps, we calculate the ranges of the bands for each $\alpha = p/1440$ with integer p between 0 and 1440. Substituting the gauge of Eq. (2) in the main text into the Hamiltonian, we obtain

$$H = \omega_0 \sum_{(n_1,n_2)} a^{\dagger}_{(n_1,n_2)} a_{(n_1,n_2)} - t \sum_{\langle (m_1,m_2),(n_1,n_2) \rangle} e^{\mp i 2\pi\alpha m_1 \delta_{m_1,n_1}\delta_{m_2,n_2\pm 1}} a^{\dagger}_{(m_1,m_2)} a_{(n_1,n_2)}, \tag{S5}$$

where (m_1, m_2) and (n_1, n_2) are the pairs of the integer indices denoting the positions of the mth and nth resonators in Eq. (1) in the main text, respectively. The index for the pseudospin σ is omitted because both pseudospins are decoupled and possess identical band structure. Due to the discrete translational symmetry of the Hamiltonian along the y-axis, we diagonalize H by introducing the following Fourier series:

$$b_{k_y,n_1} = \sum_{n_2} e^{-ik_y n_2} a_{(n_1,n_2)} \leftrightarrow a_{(n_1,n_2)} = \int_0^{2\pi} \frac{dk_y}{2\pi} e^{ik_y n_2} b_{k_y,n_1} \tag{S6}$$

for any n_1. The Hamiltonian in Eq. (S6) then becomes

$$H = \int_0^{2\pi} \frac{dk_y}{2\pi} \sum_{n_1} \left[\omega_0 - 2t\cos\left(2\pi\alpha n_1 - k_y\right) \right] b^{\dagger}_{k_y,n_1} b_{k_y,n_1} - t\left(b^{\dagger}_{k_y,n_1} b_{k_y,n_1+1} + b^{\dagger}_{k_y,n_1} b_{k_y,n_1-1} \right) = \int_0^{2\pi} \frac{dk_y}{2\pi} H_1(k_y), \tag{S7}$$

where the integrand $H_1(k_y)$ diagonalizes H. The q-periodicity of $H_1(k_y)$ due to the rationality of $\alpha = p/q$ allows another Fourier series along the x-axis:

$$c_{k_x,k_y,j}=\sum_{n_1}e^{-ik_x(n_1q+j)}b_{k_y,n_1q+j}\leftrightarrow b_{k_y,n_1q+j}=\int_0^{2\pi/q}\frac{dk_x}{2\pi/q}e^{ik_x(n_1q+j)}c_{k_x,k_y,j}\ , \tag{S8}$$

with the truncated index j = 1, 2, …, q. We finally obtain the finite-dimensional q-band Hamiltonians for the reciprocal vector $\mathbf{k} = (k_x, k_y)$ in the magnetic Brillouin zone:

$$\begin{aligned}&H_1(k_y)=\int_0^{2\pi/q}\frac{dk_x}{2\pi/q}H_2(k_x,k_y),\\&H_2(k_x,k_y)=\sum_{j=1}^{q}\left[\omega_0-2t\cos\left(2\pi\alpha j-k_y\right)\right]c^\dagger_{k_x,k_y,j}c_{k_x,k_y,j}-t\left(e^{ik_x}c^\dagger_{k_x,k_y,j}c_{k_x,k_y,j+1}+e^{-ik_x}c^\dagger_{k_x,k_y,j+1}c_{k_x,k_y,j}\right)\end{aligned}, \tag{S9}$$

where we identify q+1 with 1 for the index j. The finite-dimensional Hamiltonian $H_2(k_x, k_y)$ is diagonalized to determine the eigenfrequencies at each $\mathbf{k}$ point.

We show an examplary band structure under α = 1/4 for the $\mathbf{k}$ points in the magnetic Brillouin zone (black box in Fig. S2), which consists of four repeated regions (a unit red region in Fig. S2). The symmetry of the Hamiltonian in Eq. (S9) results in q repetitions of the band structures inside the magnetic Brillouin zone for $\alpha = p/q$, allowing the ranges of k_x and k_y values between 0 and $2\pi/q$ to fully determine each band[3]. Therefore, we discretize the $2\pi/q \times 2\pi/q$ square plaquette (the repeated part in Fig. S2) into 20 × 20 points in k_x and k_y directions to calculate the eigenfrequencies. The range of each band is then obtained from the extremums among the 400 frequency values obtained at the $\mathbf{k}$ points.

After quantifying the range of the bands, we determine whether the frequency corresponding to each pixel belongs to a band or not. For the frequencies in bandgaps, we assign the pixel the color corresponding to the gap Chern number calculated from the TKNN formula: Eq. (3) in the main text.

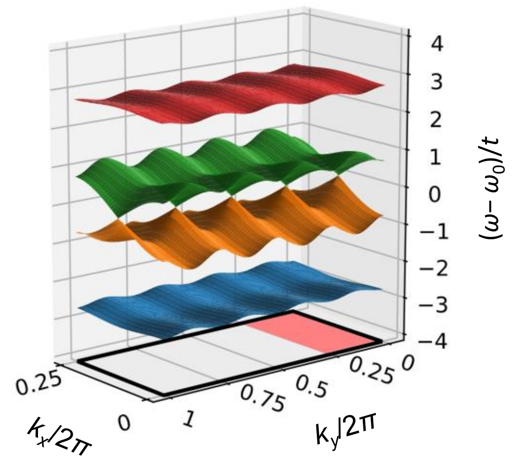

Fig. S2. Band structure example of the Hofstadter model for α = 1/4. The band structure is calculated using Eq. (S9) in the magnetic Brillouin zone (black box). The 4-fold repetition of band structure (red shaded region) is observed due to the magnetic translational symmetry.

Note S3. Butterfly wing area calculation

As the performance figure of signal transport, we numerically calculate each wing's area of the Hofstadter butterfly. We approximate the wing geometry to the hexacontagon (or 60-gon) defined with 60 vertices sampled from the wing boundary.

For the approximation, we determine the α range of each wing having a given C. Equation (3) in the main text shows that a bandgap of C is uniquely determined for every $\alpha = p/q$ if and only if $q \geq 2|C| + 1$. Therefore, the wings of C are closed at each $\alpha = p/q$ if $q \leq 2|C|$. By counting all α's where the wings are closed, we obtain the total number of wings N_C for the gap Chern number C:

$$N_C = \varphi(1) + \cdots + \varphi(2|C|), \tag{S10}$$

where $\varphi(n)$ is the Euler's phi function. The wing-closing α values also determine the exact α ranges of each wing with the gap Chern number C. For example, there are 12 wings with $C = 3$, and each of these bandgaps fills the α ranges of (0, 1/6), (1/6, 1/5), (1/5, 1/4), (1/4, 1/3), (1/3, 2/5), (2/5, 1/2), (1/2, 3/5), (3/5, 2/3), (2/3, 3/4), (3/4, 4/5), (4/5, 5/6), and (5/6, 1) (Fig. S3a).

We divide the α range of each wing into 30 subranges to construct the 60-gon that approximately surrounds the wing (Fig. S3b). If the α range is equally divided, q of the sampled α increases rapidly, which requires very large computational cost due to the enlarged supercells and higher-dimensional Hamiltonians. Therefore, we sample the α's nonuniformly with the smallest denominators (q's) within the given range of α (Fig. 3b). For instance, in the sampling of 4 values between α_1 = 2/5 and α_2 = 1/2, we sample 3/7, 4/9, 5/11, and 5/12 instead of the uniformly distributed sequence, 21/50, 11/25, 23/50, and 12/25.

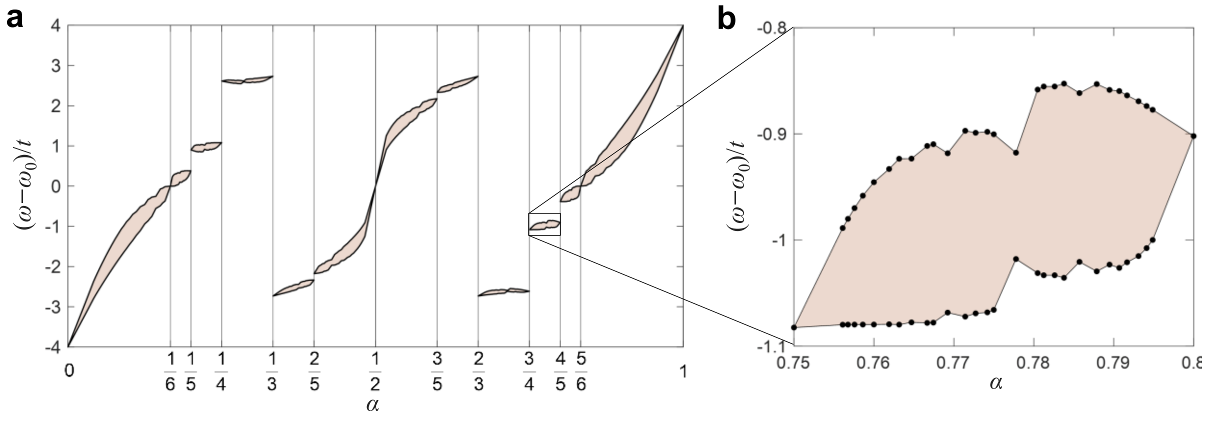

Fig. S3. Approximated wings of the Hofstadter butterfly. a, 12 wings for C = 3 according to the range of α. **b**, An inset for describing the nonuniform sampling of the α range (3/4, 4/5) to compose a 60-gon.

Note S4. System parameters of the overlapped lattice for silicon photonics

We suggest a silicon-on-chip implementation of the topological overlapped lattice by extracting the tight-binding parameters, including coupling strength t, cross-coupling κ', and system loss κ_{ext}, from the full-wave numerical analysis using the finite-difference time domain (FDTD) method[4]. From the results, we verify that κ'/t is sufficiently suppressed by adopting a conventional silicon waveguide crossing design[5], which demonstrates the validity of the ideal tight-binding modelling for the lattice overlap. Table S1 summarizes the proposed geometry and the coupling parameters.

As a practical implementation, we consider the silicon photonic slab structure, assuming the quasi-transverse electric (TE) mode operation at the telecom wavelength λ_0 = 1550 nm. To realize the lattice overlap, we employ the low-loss and low-crosstalk waveguide crossing design[5] to the junctions between lattices, where the simulation geometry and result are shown in Fig. S4. Near the target wavelength λ_0, the insertion loss and crosstalk are less than 0.15 dB and −50 dB, respectively.

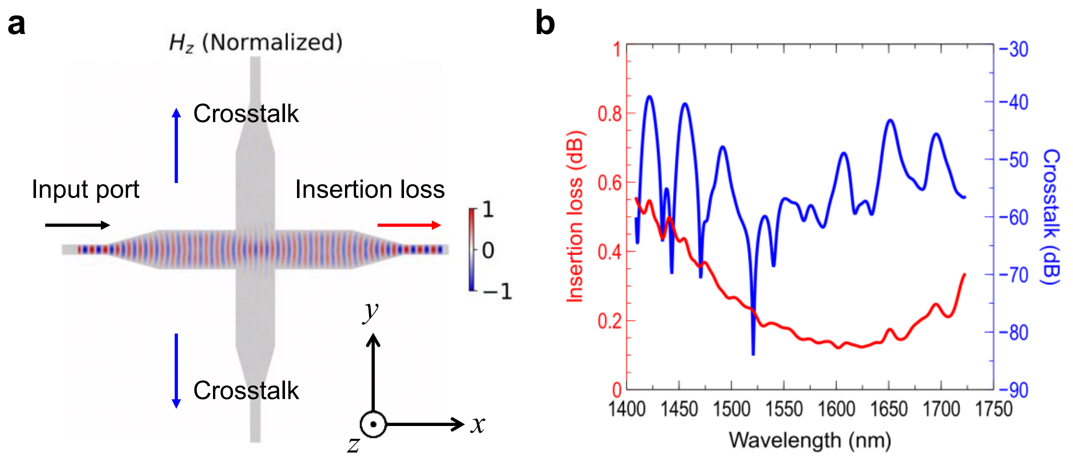

Fig. S4. Crossing geometry for lattice overlap. a, The silicon waveguide crossing[5] and propagating quasi-TE field obtained from the FDTD simulation. **b**, The simulation result for the insertion loss and crosstalk.

To investigate the impact of the crosstalk on the lattice overlap, we examine the periodic system shown in Fig. S5a. The system is modeled by the temporal CMT with the coupling strength t (solid lines in Fig. S5a), unwanted crosstalk coupling κ' (dashed lines in Fig. S5a), and internal loss κ_{ext} of each resonator. Figure S5b shows the implementation of the system's building block using ring resonators and zero-field waveguided couplings, where two waveguide loops cross each other at four points. The effective CMT model parameters can be extracted from the FDTD simulation of the geometry in Fig. S5b, as follows.

We first solve the tight-binding Hamiltonian of the CMT model to obtain an implicit dispersion relation. The model extends along the x and y directions, resulting in the two-band Hamiltonian:

$$\frac{d}{dt}\begin{bmatrix} a_{n,m} \\ b_{n,m} \end{bmatrix} = (i\omega_0 - \kappa_{\text{ext}})\begin{bmatrix} a_{n,m} \\ b_{n,m} \end{bmatrix} + it\begin{bmatrix} a_{n-1,m} + a_{n+1,m} \\ b_{n,m-1} + b_{n,m+1} \end{bmatrix} + i\kappa\begin{bmatrix} b_{n,m} + b_{n-1.m} + b_{n,m+1} + b_{n-1,m+1} \\ a_{n,m} + a_{n+1,m} + a_{n,m-1} + a_{n+1,m-1} \end{bmatrix}, \tag{S11}$$

where $a_{n,m}$ and $b_{n,m}$ are the resonator fields (Fig. S5a) and ω_0 is the resonance frequency. We apply the Bloch theorem to obtain

$$\begin{bmatrix} a_{n+1,m} \\ b_{n+1,m} \end{bmatrix} = \xi_x \begin{bmatrix} a_{n,m} \\ b_{n,m} \end{bmatrix} \quad \text{and} \quad \begin{bmatrix} a_{n,m+1} \\ b_{n,m+1} \end{bmatrix} = \xi_y \begin{bmatrix} a_{n,m} \\ b_{n,m} \end{bmatrix}, \tag{S12}$$

where ξ_x and ξ_y are the eigenvalues of the translation operators along the x and y directions, respectively. The Hamiltonian then becomes

$$\frac{d}{dt}\begin{bmatrix} a_{n,m} \\ b_{n,m} \end{bmatrix} = \begin{bmatrix} i\omega_0 - \kappa_{\text{ext}} + it(\xi_x + \xi_x^{-1}) & i\kappa'(1+\xi_x)(1+\xi_y^{-1}) \\ i\kappa'(1+\xi_x^{-1})(1+\xi_y) & i\omega_0 - \kappa_{\text{ext}} + it(\xi_y + \xi_y^{-1}) \end{bmatrix}\begin{bmatrix} a_{n,m} \\ b_{n,m} \end{bmatrix}. \tag{S13}$$

We focus on the case where $\xi = \xi_x = \xi_y$ to simplify the problem. Using the harmonic condition with the system frequency ω, we obtain

$$i\omega\begin{bmatrix}a_{n,m}\\ b_{n,m}\end{bmatrix}=\begin{bmatrix}i\omega_0-\kappa_{\text{ext}}+it(\xi+\xi^{-1}) & i\kappa'(1+\xi)(1+\xi^{-1})\\ i\kappa'(1+\xi)(1+\xi^{-1}) & i\omega_0-\kappa_{\text{ext}}+it(\xi+\xi^{-1})\end{bmatrix}\begin{bmatrix}a_{n,m}\\ b_{n,m}\end{bmatrix}. \quad \text{(S14)}$$

The Hadamard basis

$$\begin{bmatrix}c_{n,m}\\ d_{n,m}\end{bmatrix}=\frac{1}{\sqrt{2}}\begin{bmatrix}1 & 1\\ 1 & -1\end{bmatrix}\begin{bmatrix}a_{n,m}\\ b_{n,m}\end{bmatrix} \quad \text{(S15)}$$

diagonalizes the system, providing the relation between ξ and $\Delta = \omega - \omega_0$ for each band:

$$\begin{cases} i\Delta+\kappa_{\text{ext}}-it(\xi_c+\xi_c{}^{-1})-i\kappa'(1+\xi_c)(1+\xi_c{}^{-1})=0, & c_{n,m}\neq 0\\ i\Delta+\kappa_{\text{ext}}-it(\xi_d+\xi_d{}^{-1})+i\kappa'(1+\xi_d)(1+\xi_d{}^{-1})=0, & d_{n,m}\neq 0\end{cases}, \quad \text{(S16)}$$

where ξ_c and ξ_d are the ξ values corresponding to each band. The equations are equivalent to

$$\xi_c+\xi_c{}^{-1}=\frac{\Delta-2\kappa'-i\kappa_{\text{ext}}}{t+\kappa'} \quad \text{and} \quad \xi_d+\xi_d{}^{-1}=\frac{\Delta+2\kappa'-i\kappa_{\text{ext}}}{t-\kappa'}, \quad \text{(S17)}$$

which are the desired implicit dispersion relations.

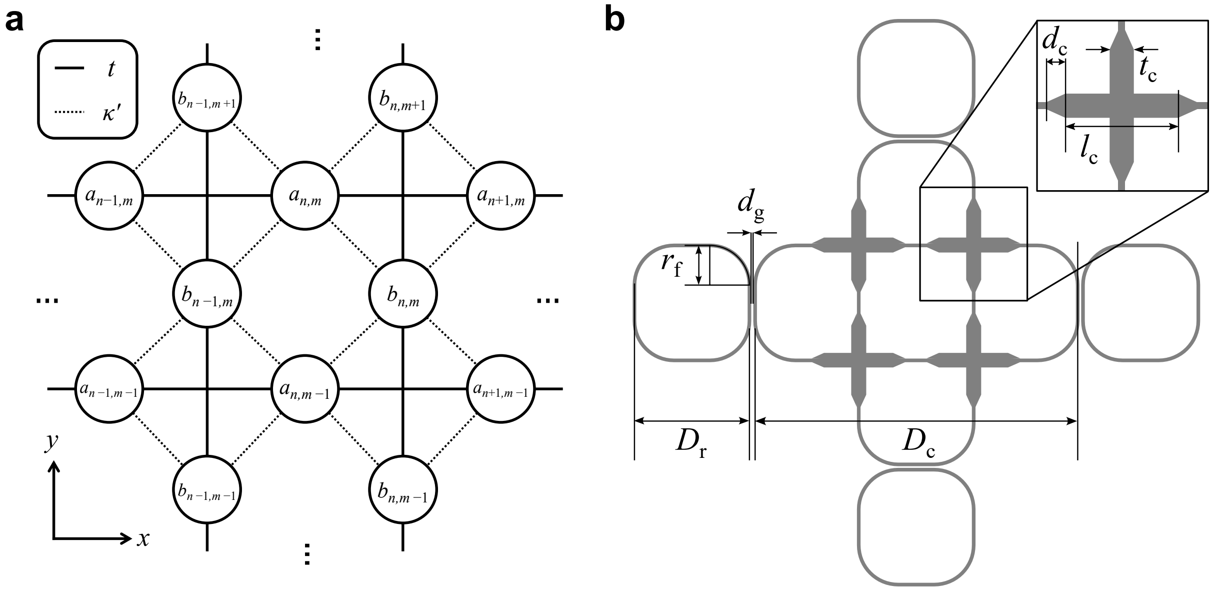

Fig. S5. Waveguide crossing in the lattice overlap. a, The structure for estimating the hopping strength t and crosstalk coupling κ' in the silicon photonics implementation. $a_{n,m}$ and $b_{n,m}$ denote the fields of resonance modes. **b**, A schematic of the unit cell implementation. D_r, D_c, d_g, d_c, r_f, l_c, and t_c represent the geometric parameters, where their values are provided in Table S1.

To numerically calculate the implicit dispersion relations, we perform the FDTD simulation on the coupling region in the blue region of Fig. S6. The simulation results in an 8 × 8 scattering matrix $S_{ij}(\omega)$, where each input field ψ_j and output field φ_i are related by $\varphi_i = \Sigma_j S_{ij}\psi_j$ (Fig. S6). We transform the scattering matrix to the transfer matrix M_c, defined as

$$\begin{bmatrix}\varphi_5 & \psi_5 & \varphi_6 & \psi_6 & \varphi_7 & \psi_7 & \varphi_8 & \psi_8\end{bmatrix}^{\mathrm{T}} = M_{\mathrm{c}}(\omega)\begin{bmatrix}\psi_1 & \varphi_1 & \psi_2 & \varphi_2 & \psi_3 & \varphi_3 & \psi_4 & \varphi_4\end{bmatrix}^{\mathrm{T}}, \quad \text{(S18)}$$

where T denotes the transpose of the vector. To construct the full transfer matrix through a unit cell of the periodic structure, the propagation through the part of resonators should be considered, as follows:

$$\begin{cases}\begin{bmatrix}\psi_1 & \varphi_1 & \psi_2 & \varphi_2 & \psi_3 & \varphi_3 & \psi_4 & \varphi_4\end{bmatrix}^{\mathrm{T}} = M_{\mathrm{r}}(\omega)\begin{bmatrix}\varphi_5' & \psi_5' & \varphi_6' & \psi_6' & \varphi_7' & \psi_7' & \varphi_8' & \psi_8'\end{bmatrix}^{\mathrm{T}} \\ M_{\mathrm{r}}(\omega) = \mathrm{diag}(e^{j\omega T}, e^{-j\omega T}, e^{j\omega T}, e^{-j\omega T}, e^{j\omega T}, e^{-j\omega T}, e^{j\omega T}, e^{-j\omega T})\end{cases}, \quad \text{(S19)}$$

where diag(**v**) is a diagonal matrix from the elements of **v** and T is the propagation time determined by the length of the resonator. The full transfer matrix is then obtained as $M(\omega) = M_c(\omega)M_r(\omega)$. The numerical dispersion relation is achieved by the following generalized eigenproblem:

$$\left|M(\omega) - \mathrm{diag}(\xi_x, \xi_x, \xi_x, \xi_x, \xi_y, \xi_y, \xi_y, \xi_y)\right| = 0. \quad \text{(S20)}$$

Same as the simplification in the CMT model, we assume $\xi = \xi_x = \xi_y$. Moreover, we employ the Hadamard basis in Eq. (S15) by applying the following basis transformation matrix V:

$$V = \frac{1}{\sqrt{2}}\begin{bmatrix}1 & 0 & 0 & 0 & 1 & 0 & 0 & 0 \\ 0 & 1 & 0 & 0 & 0 & 1 & 0 & 0 \\ 0 & 0 & 1 & 0 & 0 & 0 & 1 & 0 \\ 0 & 0 & 0 & 1 & 0 & 0 & 0 & 1 \\ 1 & 0 & 0 & 0 & -1 & 0 & 0 & 0 \\ 0 & 1 & 0 & 0 & 0 & -1 & 0 & 0 \\ 0 & 0 & 1 & 0 & 0 & 0 & -1 & 0 \\ 0 & 0 & 0 & 1 & 0 & 0 & 0 & -1\end{bmatrix}, \quad \text{(S21)}$$

where the transform matrix in the new basis becomes $M^{\mathrm{H}}(\omega) = V^{\dagger}M(\omega)V$. Neglecting the crosstalk between the modes, $M^{\mathrm{H}}(\omega)$ is a block diagonal matrix

$$M^{\mathrm{H}}(\omega) = \begin{bmatrix} M_c^{\mathrm{H}}(\omega) & O \\ O & M_d^{\mathrm{H}}(\omega) \end{bmatrix}, \tag{S22}$$

where $M_c^{\mathrm{H}}(\omega)$ and $M_d^{\mathrm{H}}(\omega)$ are 4 × 4 matrices corresponding to the basis $c_{n,m}$ and $d_{n,m}$ in Eq. (S15). We note that

$$\xi_c + \xi_c^{-1} = \frac{1}{2}\mathrm{tr}(M_c^{\mathrm{H}}(\omega)) \quad \text{and} \quad \xi_d + \xi_d^{-1} = \frac{1}{2}\mathrm{tr}(M_d^{\mathrm{H}}(\omega)), \tag{S23}$$

because the four eigenvalues of $M_c^{\mathrm{H}}(\omega)$ corresponds to forward and backward eigenvalues of the spin up and down modes, where the same holds for $M_d^{\mathrm{H}}(\omega)$.

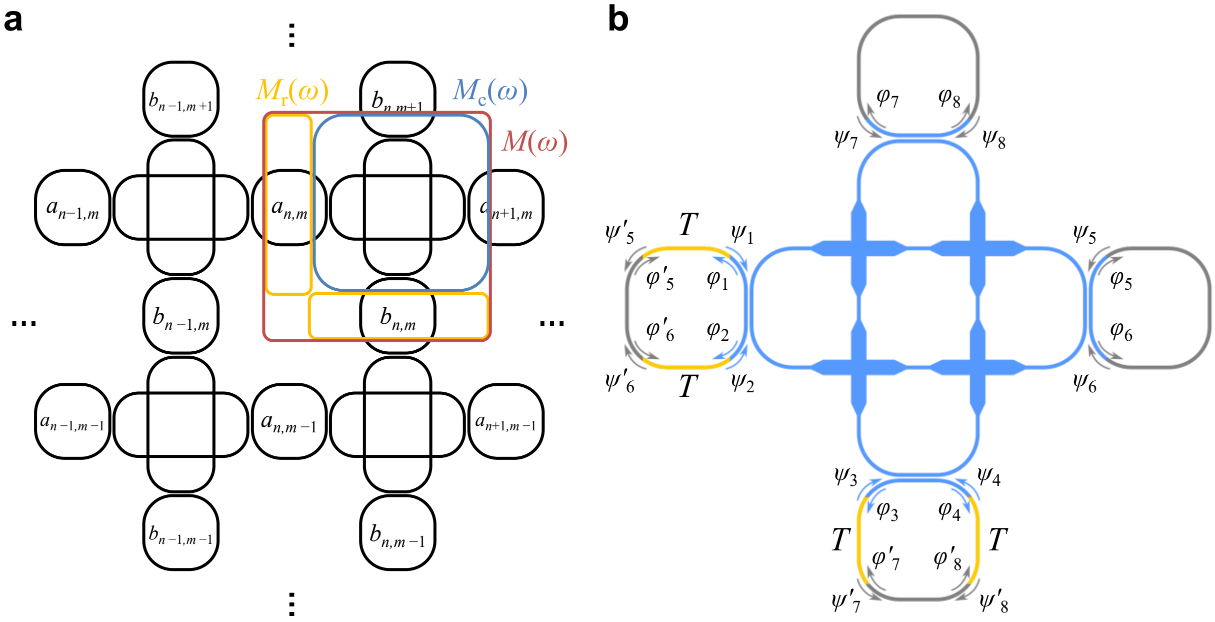

Fig. S6. a, **Silicon photonics implementation.** A schematic of silicon photonics implementation of the structure shown in Fig. S5a. $M(\omega)$ with the corresponding box denotes the transfer matrix of the unit cell. $M(\omega)$ consists of $M_c(\omega)$ and $M_r(\omega)$, which are obtained from the FDTD analysis and Eq. (S19), respectively. **b**, The unit cell structure presenting the propagating fields at several points. The propagating fields ψ_i, ψ_i', φ_i and φ_i' are connected by Eqs. (S18) and (S19).

Equation (S23) is the numerical dispersion relation based on the results of the full-wave analysis using the FDTD simulation, which is the counterpart of the analytical formulation of Eq. (S17). To compare those equations, we determine the resonator length by changing the value of T for the resonance near 1550 nm (λ_0 = 1551.5 nm) with the free spectral range around 500 GHz. Applying the designed resonator specification, we extract the coupling strength t from the slope of the real parts in $\mathrm{tr}(M_c^{\mathrm{H}}(\omega_0))/2$ and $\mathrm{tr}(M_d^{\mathrm{H}}(\omega_0))/2$ (Fig. S7a). Meanwhile, we obtain κ' and κ_{ext} from the imaginary values of $\mathrm{tr}(M_c^{\mathrm{H}}(\omega_0))/2$ and $\mathrm{tr}(M_d^{\mathrm{H}}(\omega_0))/2$ (Fig. S7b).

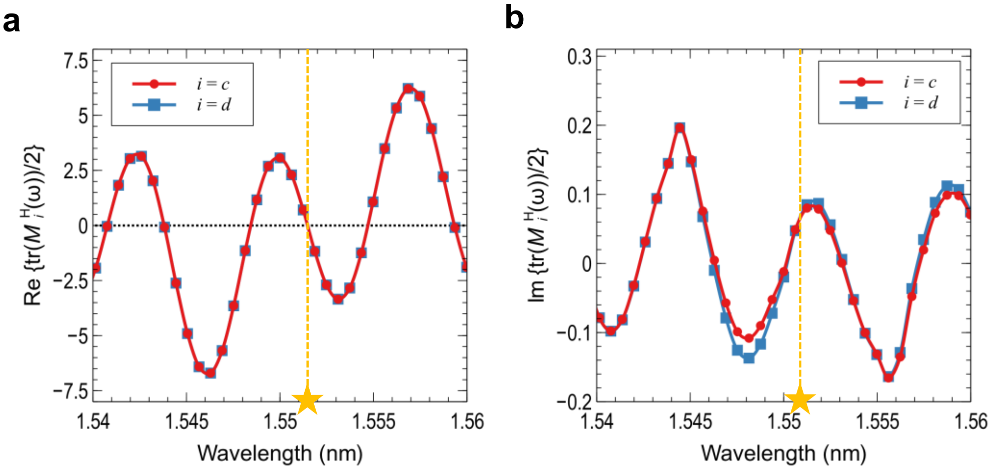

Fig. S7. System parameter extraction. The real (**a**) and imaginary parts (**b**) of the implicit dispersion relations defined in Eq. (S23). The stars denote the operation wavelength of the system (λ = 1551.5 nm), where the real-values' slopes and the imaginary values are used to extract the coupling strengths t and κ'.

Table S1. System parameters for silicon-photonics implementation.

System parameter	Symbol	Value
Geometrical parameters		
Waveguide width	w_{wg}	0.5 μm
Waveguide height	h_{wg}	0.22 μm
Resonator length	D_{r}	16 μm
Coupler length	D_{c}	44 μm
Bending radius	r_{f}	5.5 μm

Resonator-coupler gap	d_{g}	100 nm
Crossing coupler width[5]	t_{c}	1.9 μm
Crossing coupler length[5]	l_{c}	9.3 μm
Crossing coupler tapering[5]	d_{c}	2.5 μm
Wavelength and coupling parameters		
Operating wavelength	λ_0	1551.5 nm
Free spectral range	FSR	2.2 THz
Coupling strength	$\lvert t \rvert$	40 GHz
Cross-coupling	$\lvert \kappa' \rvert$	1.6 GHz
System loss	$\lvert \kappa_{\mathrm{ext}} \rvert$	3.5 GHz

Note S5. Overlapping disparate lattices

In the main text, we have focused on N-folded overlaps of identical lattices, which allow for the N multiplications of the gap Chern number C. By overlapping disparate lattices, we can design an N-folded lattice possessing an arbitrary C other than the multiples of N. Figure S8 shows a 3-folded lattice overlap, which consists of the lattices with two different magnetic fluxes α. We employ the α values (black dotted arrows in Fig. S8b) to support 4 edge modes in total at the operation frequency range (white dotted arrows in Fig. S8b), which is determined by the narrowest band gap among the three gaps of interest. To confirm the design, we examine the band structure of one-dimensional (1D) ribbon geometry of the domain (Fig. S9). To clearly reveal the edge modes resolving the degeneracy, we apply random perturbations as described in Figs. 3a,d of the main text. Figure S9b illustrates the 4 forward-propagating edge modes at the operation frequency range as expected.

In general, to construct the domain possessing the gap Chern number M by overlapping N disparate lattices, we require at least one lattice to have the gap Chern number no less than $K = \lceil M/N \rceil$ according to the pigeonhole principle. Therefore, the lattice with the gap Chern number K imposes the limit on the signal transport performance according to its corresponding bandgap.

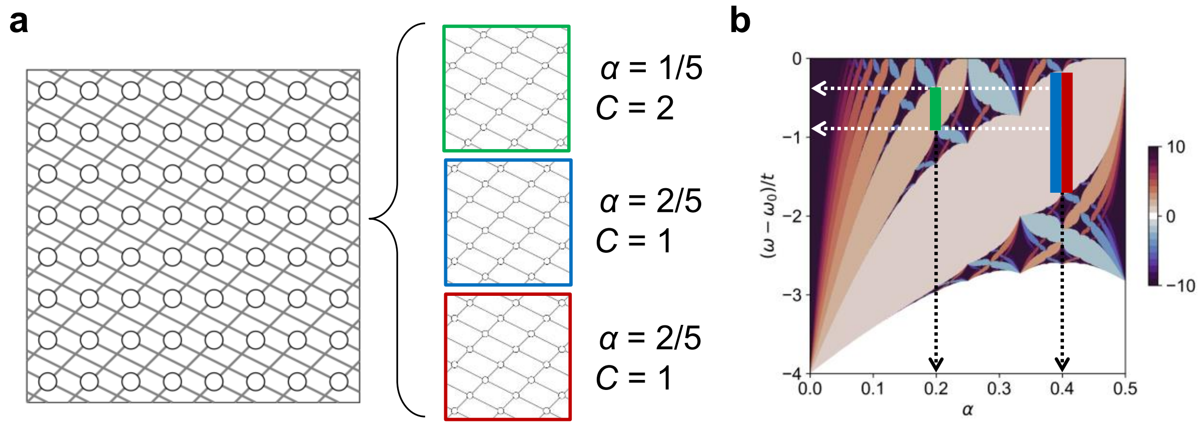

Fig. S8. The 3-folded lattice overlap. a, A schematic of the 3-folded overlapped lattice with two different α and C. Colored boxes (green, blue, and red) are depicted to distinguish each lattice component. **b**, The operation frequency range of the overlapped lattice using a part of the Hofstadter butterfly. Each colored solid line corresponds to the colored box in **a**.

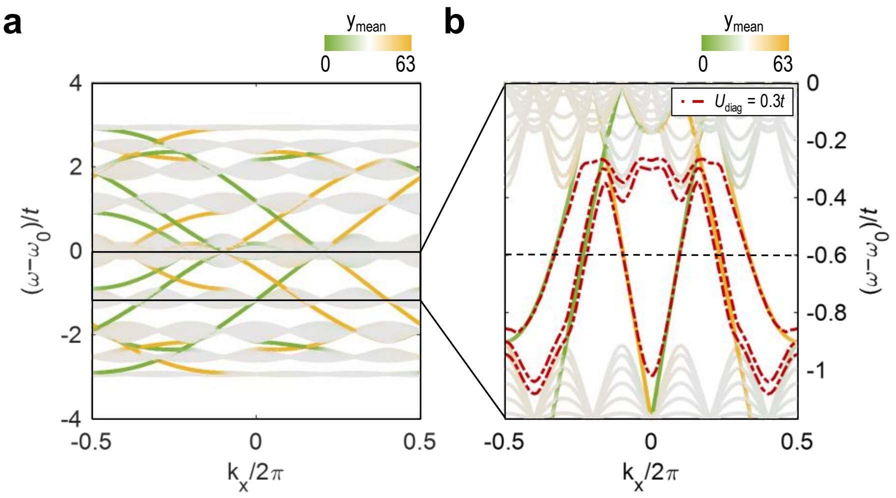

Fig. S9. The band structure of 1D ribbon geometry. a, Band structure corresponding to the system shown in Fig. S8b. **b**, The bandgap with 4 edge modes. The red dashed lines show the ensemble averaged dispersion relation under diagonal perturbations (U_{diag} = 0.3t), where the degeneracy is resolved. 500 realizations of diagonal disorder are evaluated for the ensemble average.

Note S6. Calculating band structures using ribbon geometries

To numerically calculate the band structures in Fig. 3a-c in the main text and Figs. S9 and S11, we employ the ribbon geometry: the finite system size along the *y*-axis while maintaining the translation symmetry along the *x*-axis.

We consider the geometry and hopping phases in the connections between the resonators to determine the minimum supercell that repeats in the structure. Under the phase-coded representation illustrated in Fig. S10a, Fig. S10b,c shows two exemplary designs. To minimize the *x*-axis size of the supercell, we employ the gauge varying along the *y* axis, while the gap Chern number remains unchanged under the gauge transformation. Based on the constructed supercell, we apply the Bloch boundary condition to numerically solve the finite Hamiltonian and obtain the band structure. In Fig. S11, we provide the band structure of each port shown in Fig. 4b of the main text using the ribbon geometries.

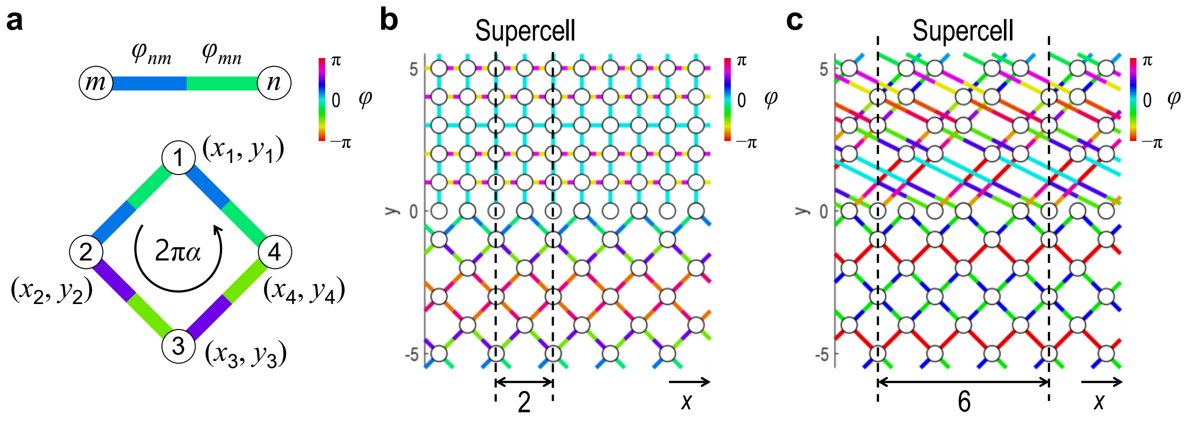

Fig. S10. Supercells in the ribbon geometries. a, The unit geometry and hopping phase between the resonators. Circles represent the resonators, and φ_{nm} and φ_{mn} are the hoping phases in the interactions between the *m*th and *n*th resonators. In the below square, four hopping phases between four resonators exhibit a synthetic flux $2\pi\alpha$. **b,c,** Two examples of ribbon geometries illustrated with the supercell. The supercells exhibit different periodicity according to the phase and connectivity between the resonators. Color bar denotes the value of hopping phases.

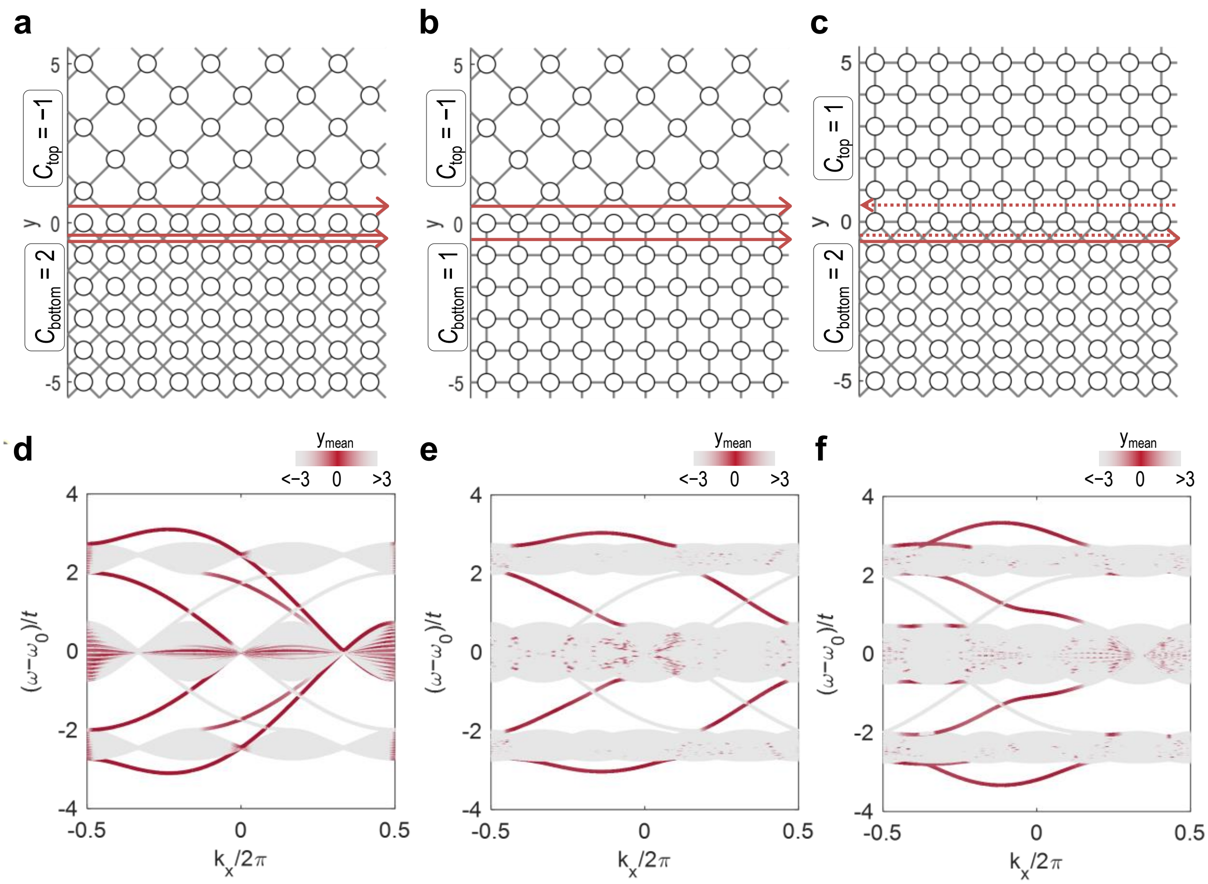

Fig. S11. Band analysis of each port in Fig. 4. a-c, Schematics of the port 1 (**a**), 2 (**b**) and 3 (**c**) in the scattering region of Fig. 4 in the main text. While arrow directions denote the propagating directions of the edge modes, a pair of the dashed arrows represents the annihilation of the edge modes due to the counter-propagation. **d-f**, Corresponding band structures for **a** (**d**), **b** (**e**) and **c** (**f**). y_{mean} denotes the *y*-axis center-of-mass values of the edge modes.

Note S7. Scattering matrix analysis

We illustrate how the open-source Python package Kwant[6] is employed to calculate the S-parameters from the photonic tight-binding model. We assume an exemplary system with a single port (Fig. S12). Specifically, we calculate the S-parameter S_{ij} between the jth input and the ith output mode, supported in the semi-infinite port, as follows.

To conduct the scattering analysis, we solve the Hamiltonian for a given ω to obtain the wavenumber. We calculate the eigenmodes of the semi-infinite port using the tight-binding model, which is described by the port matrix H_{PC} and the interconnecting matrix V_P (Fig. S12). The eigenmode ψ_P of the port satisfies $H_P\psi_P = \omega\psi_P$, where

$$H_P = \begin{bmatrix} \ddots & V_P & 0 & \cdots & 0 \\ V_P^\dagger & H_{PC} & V_P & 0 & \vdots \\ 0 & V_P^\dagger & H_{PC} & \ddots & 0 \\ \vdots & 0 & \ddots & H_{PC} & V_P \\ 0 & \cdots & 0 & V_P^\dagger & \ddots \end{bmatrix} \tag{S24}$$

is an infinite dimensional matrix and ω is the operation frequency. Due to the translational symmetry, we can employ $\psi_P = [\ldots, \lambda^{-1}\psi_{P0}, \psi_{P0}, \lambda\psi_{P0}, \ldots]$, where λ is the eigenvalue of the translation operator satisfying $\lambda = e^{ik}$ for the wavenumber k and ψ_{P0} is a finite-dimensional vector corresponding to the periodicity of the Bloch wavefunction due to the supercell. The eigenvalue equation for obtaining the wavenumber is then:

$$\left(V_P^\dagger\lambda^{-1} + H_{PC} + V_P\lambda\right)\psi_{P0} = \omega\psi_{P0}, \tag{S25}$$

which is a quadratic eigenvalue problem.

The solutions are classified according to the value of λ. A solution is amplifying or decaying with complex-valued k when $|\lambda| \neq 1$. On the other hand, in case of $|\lambda| = 1$, which allows the existence of a real-valued wavenumber k satisfying $e^{ik} = \lambda$, the solution is the propagating mode.

Before tackling the main problem with the scattering region, the periodic parts ψ_{P0} of the propagating modes are normalized with the power flow:

$$P = 2\,\mathrm{Im}\left(\lambda \psi_{P0}^{\dagger} V_P \psi_{P0}\right). \tag{S26}$$

$P = \pm 1$ then confirms that each propagating mode carries the same amount of power.

The full eigenvalue equation of the system including the scattering region is

$$\begin{bmatrix} \ddots & V_P & 0 & 0 \\ V_P^{\dagger} & H_{PC} & V_P & 0 \\ 0 & V_P^{\dagger} & H_{PC} & V_C \\ 0 & 0 & V_C^{\dagger} & H_S \end{bmatrix} \begin{bmatrix} \vdots \\ \psi_2 \\ \psi_1 \\ \psi_0 \end{bmatrix} = \omega \begin{bmatrix} \vdots \\ \psi_2 \\ \psi_1 \\ \psi_0 \end{bmatrix}, \tag{S27}$$

where V_C connects the port and the scattering region, H_S is the Hamiltonian of the scattering region, and the eigenmode consists of the finite-dimensional vectors ψ_n for the integer $n \geq 0$ (Fig. S12). The equations of the lowest two rows are

$$\begin{cases} \omega\psi_0 = V_C^{\dagger}\psi_1 + H_S\psi_0 \\ \omega\psi_1 = V_P^{\dagger}\psi_2 + H_{PC}\psi_1 + V_C\psi_0 \end{cases}. \tag{S28}$$

Cancelling out ψ_0, we obtain

$$\left[\left(\omega I - H_{PC}\right) - V_C\left(\omega I - H_S\right)^{-1} V_C^{\dagger}\right]\psi_1 = V_P^{\dagger}\psi_2, \tag{S29}$$

where I is the identity matrix. The other equations obtained from the upper rows of the matrix equation Eq. (S27) restrict ψ_1 and ψ_2 to be the linear combinations of the eigenmodes supported by the port:

$$\begin{cases} \psi_1 = \sum_i S_{ij}\psi_{P0,i}^{\text{out}}\lambda_i^{\text{out}} + \sum_k U_{kj}\psi_{P0,k}^{\text{decay}}\lambda_k^{\text{decay}} + \psi_{P0,j}^{\text{in}}\lambda_j^{\text{in}} \\ \psi_2 = \sum_i S_{ij}\psi_{P0,i}^{\text{out}} + \sum_k U_{kj}\psi_{P0,k}^{\text{decay}} + \psi_{P0,j}^{\text{in}} \end{cases}, \tag{S30}$$

where $\psi_{P0,j}^{\text{in}}$, $\psi_{P0,i}^{\text{out}}$, $\psi_{P0,\text{k}}^{\text{decay}}$, λ_j^{in}, λ_i^{out}, and λ_k^{decay} are the eigenvectors and eigenvalues obtained from Eq. (S25), corresponding to the propagating inward, outward, and decaying solutions

according to the indices, and S_{ij} and U_{ij} are the scattering parameters from the jth input to the ith propagating and evanescent modes of the port, which are unknown linear coefficients to be determined. We note that S_{ij} is obtained by plugging Eq. (S30) into Eq. (S28).

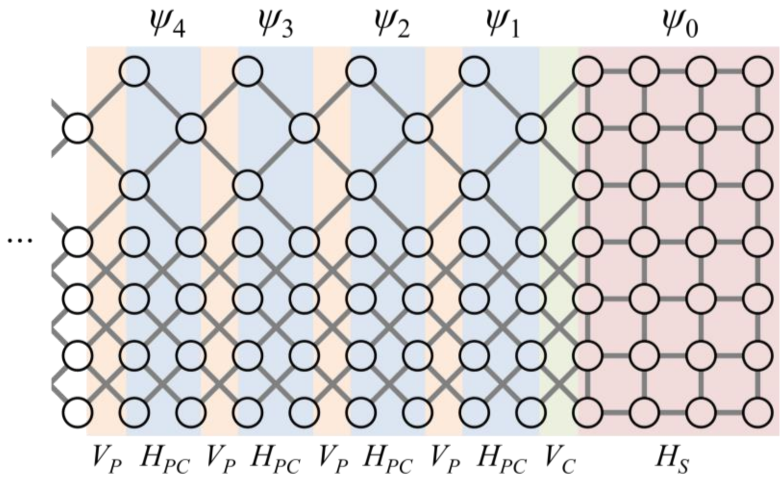

Fig. S12. Calculation of the *S*-parameters from the tight binding model. The exemplary semi-infinite system with a single port. H_{PC}, H_S, V_P, V_C, and ψ_n are the port matrix, the Hamiltonian of the scattering region, the interconnecting matrix between the ports, the interconnecting matrix between the port and the scattering region, and the eigenmodes of the ports and the scattering region, respectively.

Note S8. Robustness against diagonal and hopping-phase disorders

To evaluate the topological protection of the beam splitting functionality, we examine the transmittances against different types of disorder. Figures S13a and S13b show the transmittance of the incoherent incidence for 50 realizations of diagonal and hopping-phase disorder, respectively. The diagonal disorder is defined with the application of uniform random perturbation $[-U_{\mathrm{diag}},+U_{\mathrm{diag}}]$ to the resonance frequency of each resonator. The hopping-phase disorder is defined with the uniform random perturbation $[-2\pi U_{\mathrm{phase}},+2\pi U_{\mathrm{phase}}]$ applied to the phase of the hopping interaction between resonators.

In diagonal disorder example, although the overall optical functionality is maintained due to topological protection, the frequency range of the 2:1 beam splitting is gradually reduced , which is attributed to the Anderson localization[7] (Fig. S13a). In contrast, Fig. S13b illustrates the simultaneous presence of fully functional and malfunctioning operations in hopping-phase disorder realizations even for strong disorder (U_{phase} = 2.0). Such highly robust topologically protected edge modes can be understood with the maintenance of topological invariants despite strong phase and flux fluctuations, which is in line with topological protection in amorphous models[8-10]. Notably, $\Delta\alpha$ in Fig. 1b of the main text ultimately limits the maximal strength of hopping-phase disorder to guarantee the perfect optical functionality within the target frequency range in every disorder realization. For the operation frequency targeted in Fig. 4 of the main text ($\omega = \omega_0 - 1.5t$), we estimate $U_{\mathrm{phase}} \leq \Delta\alpha/4 = 0.07$ as the acceptable hopping-phase disorder, which coincides with the results shown in Fig. S13b.

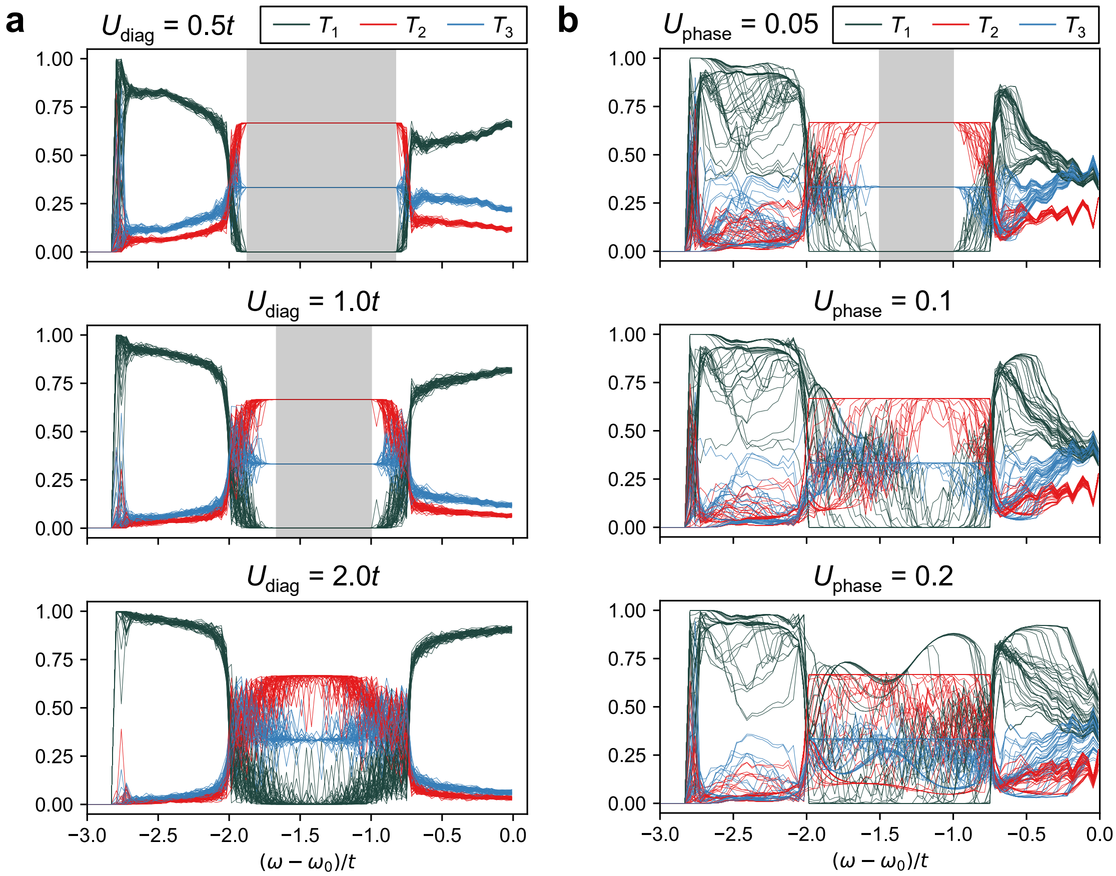

Fig. S13. Beam splitting of incoherent light against diagonal and hopping-phase disorder. a, The transmittances for the incoherent incidence against diagonal disorder with different U_{diag}. **b**, The transmission for the incoherent incidence against hopping-phase disorder with different U_{phase}. For each type of disorder with varying U_{diag} and U_{phase}, 50 random realizations are estimated, where each line in **a** and **b** denotes a realization. The shaded regions denote the frequency ranges of perfect beam splitting operation against disorder.

Note S9. Performance limitations in lattice sizes and bandwidths

According to the tight-binding model in Eq. (1) in the main text, the device bandwidth using our lattice overlap model is primarily governed by the hopping strength *t*. This ideal condition is valid because the value of *t* (= 40 GHz) is much smaller than the signal bandwidths of other optical elements, such as adiabatic waveguide crossing (Fig. S4b). The dependency of the bandwidths on hopping strength is illustrated in Fig. S14, comparing the conventional Hofstadter lattice (Fig. S14a,b,e,f) and our overlapped lattice (Fig. S14c,d,g,h) for different values of hopping strengths *t*/2 and *t*. As demonstrated, the bandwidth is directionally proportional to the hopping strength, at least, in an ideal condition.

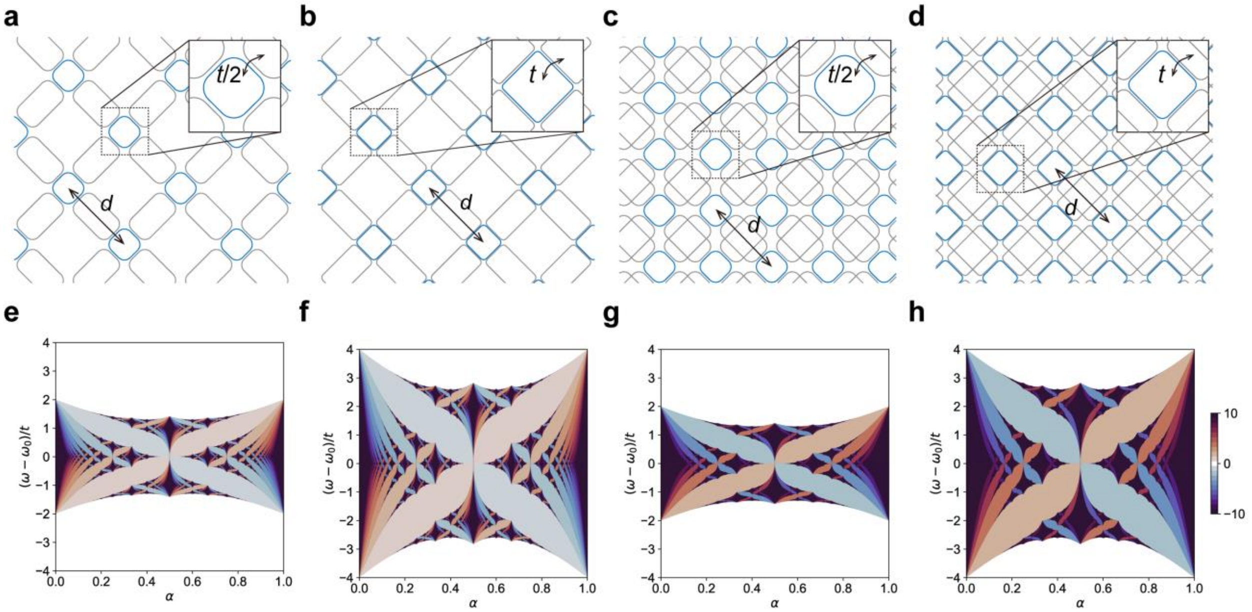

Fig. S14. Lattice bandwidths governed by hopping constants. a-d, The lattice structures and **e-h,** corresponding butterflies for the conventional Hofstadter model (**a,b,e,f**) and the lattice overlap model (**c,d,g,h**) with different values of hopping strength: *t*/2 (**a,c,e,g**) and *t* (**b,d,f,h**). In **a-d**, blue rounded squares illustrate the resonators. The insets in **a-d** describe the change of the gap between the resonator and waveguide coupler for controlling the evanescent coupling. The symbol '*d*' in **a-d** denotes the distance between connected resonators.

However, in our lattice overlap model, the performance is limited due to the system size issue. Figure S15 shows the relationship between the number of lattice overlaps N and the characteristic length scale d: the distance between the resonator connected through a waveguide coupler. As shown in Fig. S15a,b, the averaged characteristic length increases proportionally to $\sim N^{1/2}$ even when neglecting the spatial length of the adiabatic waveguide crossing structure. In a practical realization, this footprint issues becomes worsen. We note that the entire size of the lattice is governed by waveguide crossing structures due to their tapering shapes for wide operation bandwidths. Because the number of the necessary crossing is proportional to the overlap number N, the averaged characteristic length becomes $\sim N$. This enlarged system footprint enforces the degradation of the integration level of the lattice (Fig. S16c). Importantly, to maintain the lattice size, a more compact design of waveguide crossing structures is necessary, such as impedance matching techniques, though such approaches typically lead to the significant degradation of operation bandwidths.

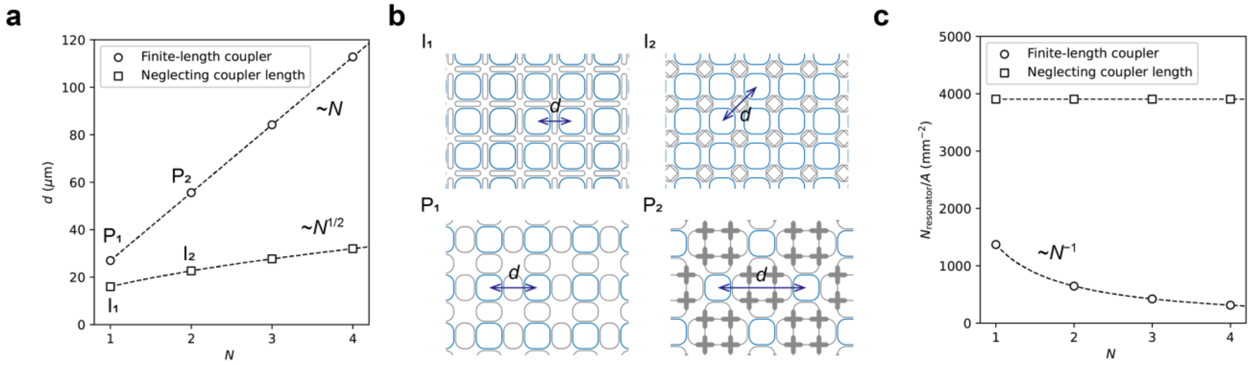

Fig. S15. Characteristic length scales and integration. a, The characteristic length d as a function of the overlap number N. **b,** The schematics for illustrating the characteristic length at each lattice overlap number. The symbol 'I_N' (or 'P_N') represents ideal (or practical) cases, neglecting (or considering) the coupler length. In **b**, blue rounded squares illustrate the resonators. **c,** The variation of the lattice integration with respect to N. $N_{resonator}$ depicts the number of resonators inside the unit cell, and A denotes the unit cell area.

Note S10. Geometry of the overlapped Haldane lattice

Figure S16 shows a possible geometry of the overlapped Haldane lattice. We properly deformed and translated the Haldane lattice as shown in Fig. S16b to satisfy the criteria of the lattice overlap in the main text. The band structure of the lattices in Fig. S16a and S16b can be identically designed because the non-resonant waveguide loops comprising the connections allow the distance-independent coupling between the resonators. The overlapped lattice supports the Chern number equivalent to the sum of the Chern numbers of composing lattices.

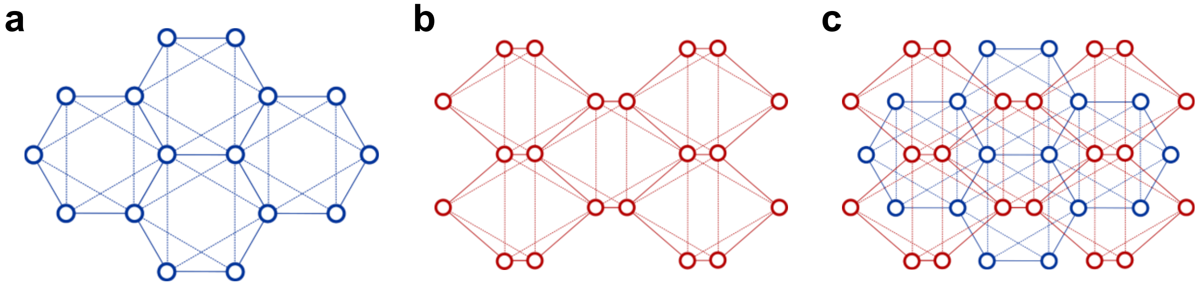

Fig. S16. Overlapping two Haldane lattices. a,**b**, Two Haldane lattices with nearest-neighbor (solid lines) and next-nearest-neighbor (dotted lines) interactions between the resonators (circles). **b** describes the deformed lattice of the Haldane lattice for satisfying the design criteria of the lattice overlap, while maintaining the original Hamiltonian. **c**, A possible network structure of the overlapped Haldane lattice.

Note S11. Competition between different orders of interactions in the overlapped lattice

We examine the competition between the nearest-neighbor (NN) and next-nearest-neighbor (NNN) interactions using the overlapped lattice platform (Fig. S17a). The leftmost structure is the overlapped lattice with two wide bandgaps (Fig. 3a,d of the main text), which possesses only the NNN interactions for the flux α = 1/4 per the diamond-shaped unit cell from the corresponding vector potential $\mathbf{A} = (0, 2\pi x/8, 0)$.

We increase the NN interaction strength from zero ($t_{NN} = 0$) to twice the strength of the NNN interactions ($t_{NN} = 2t_{NNN}$), which leads to the mixing of two degenerate bands. We investigate the evolutions of the band structures in two cases: the NN interactions without (Fig. S17b) and with (Fig. S17c) the hopping phase determined by the vector potential **A**. Starting from the same band structures, the two evolutions dramatically diverge. While the 2D square lattice only with zero-phase NN interactions supports a single band ranging from $\omega_0 - 4t_{NN}$ to $\omega_0 + 4t_{NN}$, the bandgaps in Fig. S17b are closed near $t_{NN} = 0.26t_{NNN}$. In contrast, the NN interactions with nonzero phases maintain the gap openings during the mixing of the degenerate bands, leading to a variety of topological band structures that converge to the single Hofstadter lattice with α = 1/8 when $t_{NN}/t_{NNN} \gg 1$.

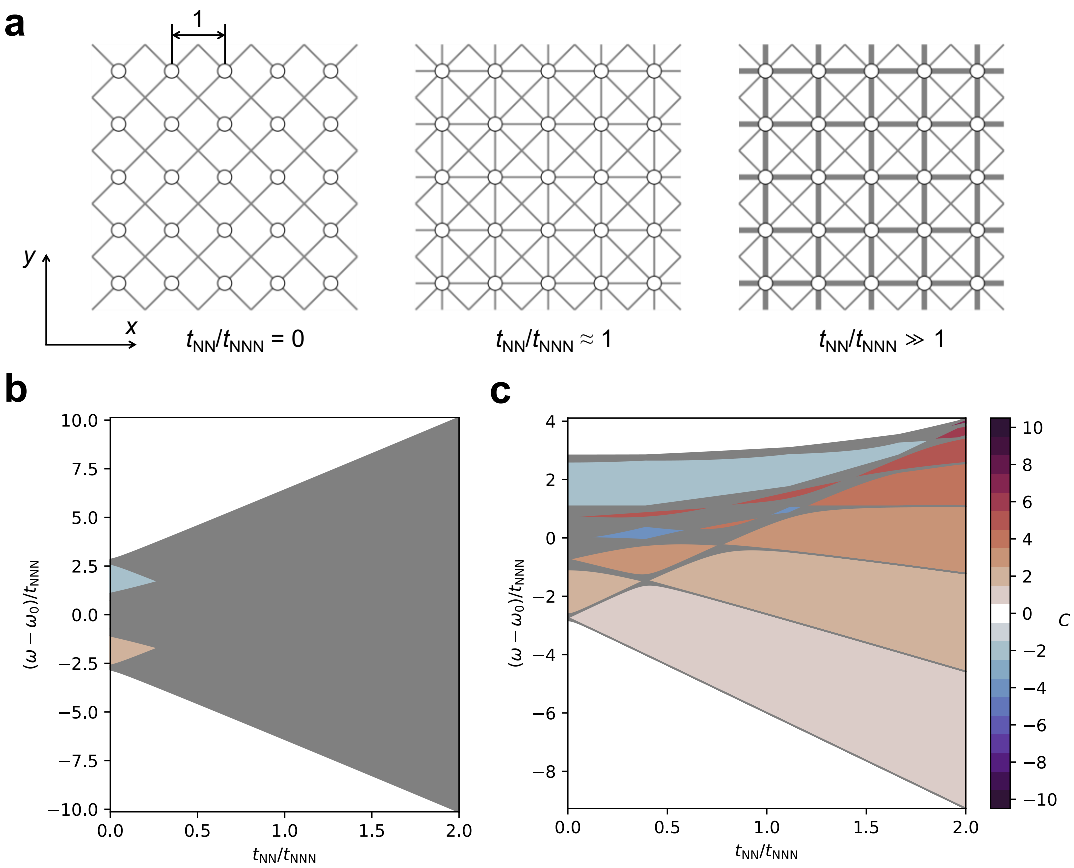

Fig. S17. Competition of NN and NNN interactions in the doubly overlapped Hofstadter lattice. **a**, Different strengths of NN interactions imposed on the overlapped lattice. **b**,**c**, Evolution of the band structure under increasing NN interactions **b**, without and **c**, with hopping phases. We color the band in gray and the topologically nontrivial bandgaps with colors corresponding to the gap Chern numbers *C*.

References

1 Hafezi, M., Demler, E. A., Lukin, M. D. & Taylor, J. M. Robust optical delay lines with topological protection. *Nat. Phys.* **7**, 907-912 (2011).

2 Yu, S., Piao, X. & Park, N. Topological hyperbolic lattices. *Phys. Rev. Lett.* **125**, 053901 (2020).

3 Fradkin, E. *Field Theories of Condensed Matter Physics*. Second edn (Cambridge University Press, 2013).

4 *Flexcompute, Inc. Tidy3D. https://www.flexcompute.com/tidy3d/solver/.*

5 Johnson, M., Thompson, M. G. & Sahin, D. Low-loss, low-crosstalk waveguide crossing for scalable integrated silicon photonics applications. *Opt. Express* **28**, 12498-12507 (2020).

6 Groth, C. W., Wimmer, M., Akhmerov, A. R. & Waintal, X. Kwant: a software package for quantum transport. *New J. Phys.* **16**, 063065 (2014).

7 Anderson, P. W. Absence of diffusion in certain random lattices. *Phys. Rev. Lett.* **109**, 1492-1505 (1958).

8 Marsal, Q., Varjas, D. & Grushin, A. G. Topological Weaire-Thorpe models of amorphous matter. *Proc. Natl. Acad. Sci. USA* **117**, 30260-30265 (2020).

9 Jia, Z. *et al.* Disordered topological graphs enhancing nonlinear phenomena. *Sci. Adv.* **9**, eadf9330 (2023).

10 Sahlberg, I., Ivaki, M. N., Pöyhönen, K. & Ojanen, T. Quantum Hall effect and Landau levels without spatial long-range correlations. *Phys. Rev. Res.* **5**, 033218 (2023).
